# The Great Melting Pot. Common Sole Population Connectivity Assessed by Otolith and Water Fingerprints

**DOI:** 10.1371/journal.pone.0086585

**Published:** 2014-01-27

**Authors:** Fabien Morat, Yves Letourneur, Jan Dierking, Christophe Pécheyran, Gilles Bareille, Dominique Blamart, Mireille Harmelin-Vivien

**Affiliations:** 1 Aix Marseille Université, CNRS/INSU, IRD, Mediterranean Institute of Oceanography (MIO) UM 110, Marseille, France; 2 Université de Toulon, CNRS/INSU, IRD, Mediterranean Institute of Oceanography (MIO) UM 110, La Garde, France; 3 Université de la Nouvelle-Calédonie, Laboratoire LIVE et LABEX « Corail », BP R4, Nouméa, New Caledonia; 4 Helmholtz Centre for Ocean Research (GEOMAR), Kiel, Germany; 5 Université de Pau et des Pays de l'Adour, LCABIE, UMR 5254 CNRS/IPREM, Pau, France; 6 Laboratoire des Sciences du Climat et de l'Environnement, UMR 8212 CEA/CNRS/UVSQ, Gif-sur-Yvette, France; University of Canterbury, New Zealand

## Abstract

Quantifying the scale and importance of individual dispersion between populations and life stages is a key challenge in marine ecology. The common sole (*Solea solea*), an important commercial flatfish in the North Sea, Atlantic Ocean and the Mediterranean Sea, has a marine pelagic larval stage, a benthic juvenile stage in coastal nurseries (lagoons, estuaries or shallow marine areas) and a benthic adult stage in deeper marine waters on the continental shelf. To date, the ecological connectivity among these life stages has been little assessed in the Mediterranean. Here, such an assessment is provided for the first time for the Gulf of Lions, NW Mediterranean, based on a dataset on otolith microchemistry and stable isotopic composition as indicators of the water masses inhabited by individual fish. Specifically, otolith Ba/Ca and Sr/Ca profiles, and δ^13^C and δ^18^O values of adults collected in four areas of the Gulf of Lions were compared with those of young-of-the-year collected in different coastal nurseries. Results showed that a high proportion of adults (>46%) were influenced by river inputs during their larval stage. Furthermore Sr/Ca ratios and the otolith length at one year of age revealed that most adults (∼70%) spent their juvenile stage in nurseries with high salinity, whereas the remainder used brackish environments. In total, data were consistent with the use of six nursery types, three with high salinity (marine areas and two types of highly saline lagoons) and three brackish (coastal areas near river mouths, and two types of brackish environments), all of which contributed to the replenishment of adult populations. These finding implicated panmixia in sole population in the Gulf of Lions and claimed for a habitat integrated management of fisheries.

## Introduction

One of the most challenging problems in marine ecology is to quantify the scale and magnitude of individual dispersion between populations and life stages [1]. Most vagile coastal marine fish species achieve dispersion principally during their larval and juvenile lives, and show relatively small scale movements when adults [2] (but see[3]). Eggs and first larval stages are often considered globally passive and their dispersion mostly depends on currents. However, several authors have demonstrated that larval stages may present an active orientation and swimming towards a well defined habitat [4], [5], possibly through perception of chemical cues [6]. In addition, according to larval life duration and behaviour, larvae of some species can disperse over long distances up to more than 100 km before benthic settlement and nursery colonisation [7], [8]. The end of the juvenile stage, when individuals migrate out off nurseries to recruit into adult populations, represents a second dispersion opportunity for many species [9]. The success of both the larval and the juvenile stages thus contribute to the renewal of adult populations. The estimation of rates, temporal scales and spatial structures of individual exchanges between life stages or populations, known as ecological connectivity [10], is therefore of prime importance to understand the replenishment of exploited fish populations, notably in a general context of stock declines [11], [12].

The study of connectivity and fish stock structure has benefited from technological advances particularly through population genetics [13], artificial tagging [14], otolith isotopy [15] and otolith microchemistry [16], [17]. Otoliths, which are small calcified structures in the inner ear of teleost fish are important for several physiological processes such as mecano-reception, equilibrium and audition [18]. They exhibit three key features of great use for biological and ecological studies, namely, (1) continuous growth by accretion of daily and annual layers from the birth to the death of fish, (2) metabolic inertness (i.e., newly-deposited material is neither resorbed nor reworked after deposition) [19], [20], and (3) the trace elements uptake during the otolith growth reflects the physical and chemical characteristics of the environment (“elemental fingerprint”) [21], although with significant physiological regulation [22], [23]. Due to these characteristics, otoliths can be used as life-history records reflecting habitat changes over the lifetime of individual fish. Such specificities have been widely used in the study of connectivity between populations and/or life stages of various fish species [17], [24], [25].

**Figure 1:**
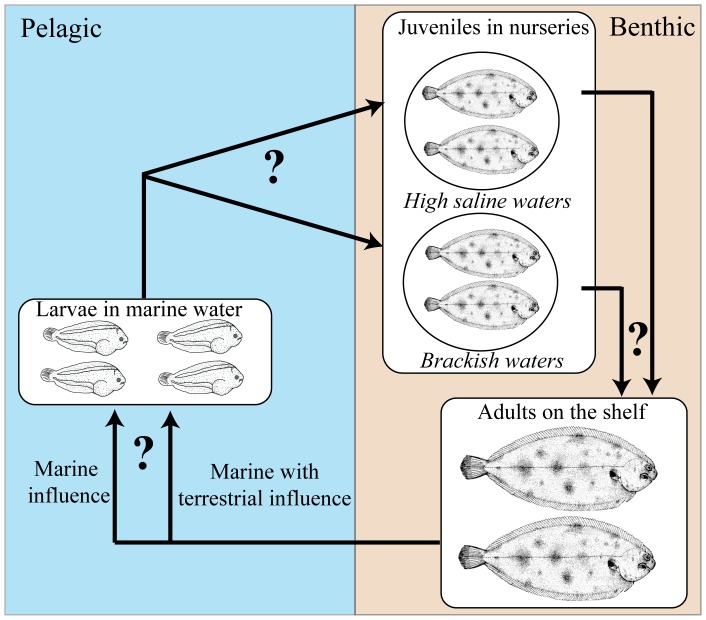
The common sole has a complex life cycle, with a pelagic marine larval stage and a benthic juvenile stage in nurseries (estuaries, shallow marine waters, coastal lagoons), before settling in benthic deep marine waters as adults. In this study, otolith and water microchemistry and isotopic signatures allowed a first estimate of the connectivity between the different life stages of the sole in the Gulf of Lions, an essential step for understanding of population dynamics of this species.

In the Eastern Atlantic Ocean, North Sea and the Mediterranean Sea, the common sole, *Solea solea* (Linnaeus, 1758), is an important commercial flatfish. In the Gulf of Lions (hereafter GoL) (NW Mediterranean Sea), annual common sole catches dropped from 520 tonnes in 1990 to 130 tonnes in 2009 (http://www.fao.org [26]). The general life cycle of the common sole, with a complex ontogenetic habitat shift [27] from a pelagic marine larval stage to a juvenile stage (young-of-the-year, hereafter YOY) in coastal nurseries (estuaries, shallow marine waters and coastal lagoons) to adult life in benthic marine habitats on the continental shelf, has been described [28], [29], [30]. At the same time, quantitative understanding of the importance of the respective habitats within life stages, and the connectivity between larval, juvenile and adult stages in the GoL is lacking. Our goal here was to address these knowledge gaps using the records of past and present habitat use inscribed as microchemical and isotopic signatures of otoliths. Specifically, to characterise the influences undergone by fish when they were larvae, signatures of the larval life of YOY retrieved from otoliths were compared to ambient water signatures. Secondly, to assess the importance of different possible nurseries for adult population replenishment, typical imprints of different nursery types were characterised based on signatures and otolith growth of the juvenile life stage of YOY from these locations (Figure 1). For each adult, the signatures of the otolith portion reflecting the larval life stage were then compared to these typical imprints to classify its larval nursery use. Similarly, the signatures and otolith growth corresponding to the juvenile life stage of adults were compared to same parameters in the juvenile life stage of YOY to classify their juvenile nursery use. The majority of published studies to date have used multi-elemental otolith fingerprints to discriminate and classify individuals from predefined habitats (e.g. [25], [31]). Both microchemistry and stable isotope composition of otoliths are linked to characteristics of water masses inhabited by fish. They were used here to elucidate the habitat used by fish during their life cycle. The originality of our study is based on the analysis of individual chemical profiles plus their isotopic signatures to characterise the habitats used during the different life stages. This combined approach allowed the quantification of the relative contributions of the different habitats used by the common sole during earlier life stages to the next older stage (larvae to juveniles, juveniles to adults).

## Materials and Methods

### Ethics statement

Fish, collected during the oceanographic IFREMER MEDITS surveys (oceanographic vessel “Europe”) or bought from local fishermen (for Mauguio and Thau lagoons), were immediately put on ice (<5°C) to be anesthetized and minimize fish suffering. Afterward, they were frozen at -20°C for conservation before dissection and otolith extraction. Protocols used during MEDITS surveys were validated by the steering committee of the international program according to European regulations. In this study no protected species was collected.

### Sampling and otolith preparation

**Figure 2:**
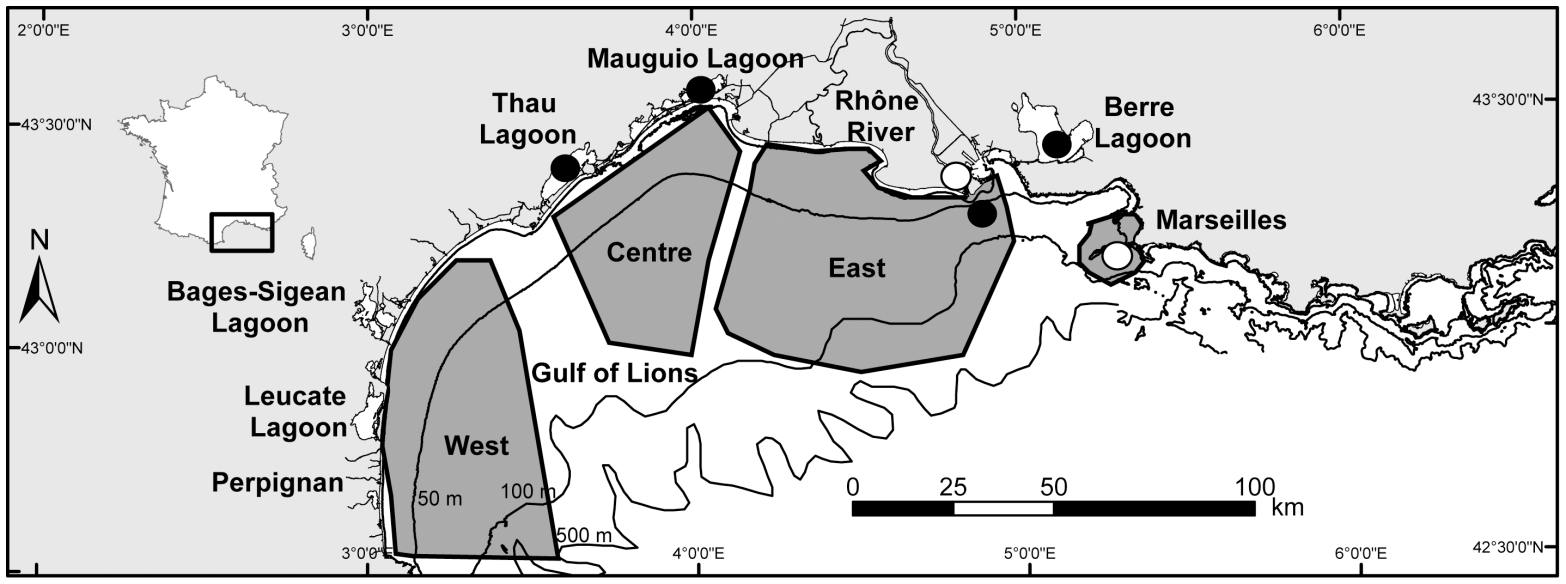
Location of the study sites in the Gulf of Lions (North-Western Mediterranean Sea). The four dark-grey areas (West, Centre, East and Marseilles) represent the sampling locations of adults, the black points the sampling locations of YOY and the white points the sampling locations of water. For specific sample size see Table 1.

**Figure 3:**
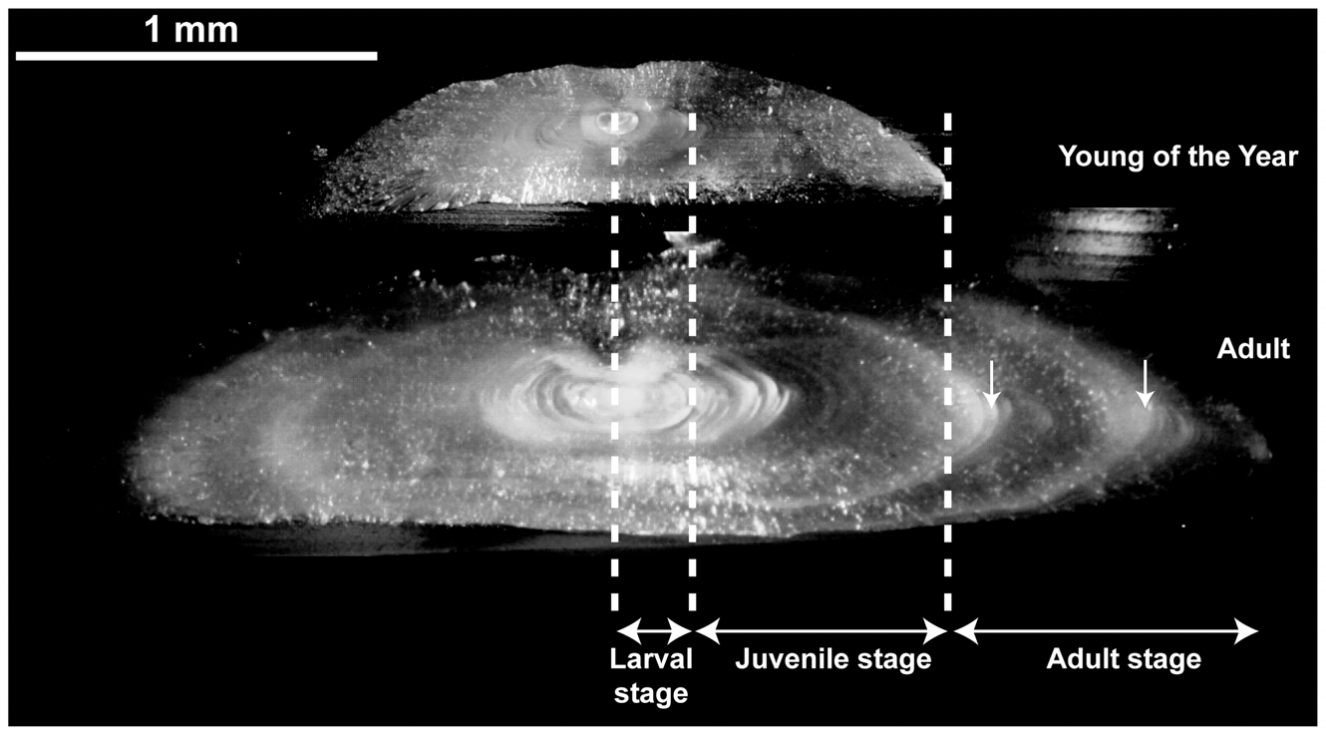
Representation of life stages on inner sections of otoliths of YOY and adults. White large rings represent the annual marks (vertical arrows).

A total of 80 common sole adults (2 and 3 years old) were collected by trawling in the West, Centre and East parts of the GoL and near Marseilles, during the oceanographic Ifremer MEDITS campaign in spring 2008 and by fishermen in fall 2008 (Figure 2, Table 1). In addition, 113 YOY were sampled in different nurseries shortly before moving back out to sea (16 near the Rhône River in fall 2000, 16 in Berre lagoon in fall 2008, 32 in Mauguio lagoon in fall 2004 and 2008 and 49 in Thau lagoon in fall 2003, 2004 and 2008; Figure 2). Left otoliths were extracted with non-metallic forceps, cleaned and dried. Otoliths were then embedded in araldite 2020 resin (Escil Chassieu, France), cut on a transversal plan to expose the core and polished (average thickness ∼350 µm). Finally otoliths were rinsed with milliQ water and dried prior to laser ablation or stable isotopic analyses. For each sample, the larval stage was defined as the period between hatching and metamorphosis [32], and the juvenile stage between metamorphosis and the age of one year, whereas older individuals (2+ and 3+) were defined as adults (Figure 3).

**Table 1:** Number (N) of common sole adults analysed in the two age-classes in this study sampled in the Gulf of Lions.

Site	Age	Year of larval life	N	Size range (mm)
Gulf of Lions West	2+	Winter 2005–2006	12	265–372
	2+	Winter 2004–2005	8	257–324
	3+	Winter 2004–2005	15	289–376
Gulf of Lions Centre	2+	Winter 2005–2006	10	260–347
	3+	Winter 2004–2005	4	273–354
Gulf of Lions East	2+	Winter 2005–2006	10	279–360
	2+	Winter 2004–2005	5	265–376
	3+	Winter 2004–2005	6	292–365
Marseilles	2+	Winter 2005–2006	4	263–286
	2+	Winter 2004–2005	5	259–364
	3+	Winter 2004–2005	1	280

### Water sampling and analysis

Water samples were collected at subsurface four times in winter (January-February) at two sites, the Bay of Marseilles and Port-Saint-Louis du Rhône (Figure 2) to obtain information on seawater and Rhône River water chemical characteristics respectively. Since the damming of the Nile in 1970, the Rhône River is the river with the largest discharge (mean of 1 700 m^3^ s^-1^, ranging from <500 m^3^ s^−1^ in summer to up to 10–11 000 m^3^ s^−1^ during the flooding period [33]), draining into the Mediterranean Sea, and dominates runoff in the Gulf of Lions. The Rhône River exports on average 6.2×10^6^ t a^−1^ of terrestrial particulate material and is estimated to drive ∼50% of the phytoplankton production of the GoL [34]. An exceptional 100-year return flood event occurred in December 2003, with a maximum discharge observed the 3^rd^ December 2003 (∼12 000 m^3^ s^−1^ [35]). During this event, 5.4×10^6^ t of suspended particulate matter and a large quantity of dissolved elements were transported to the GoL [35], [36].

Sample and analysis processing followed Tabouret et al. [37]. Briefly, for each sample, 50 mL of water were filtered through a 0.45 µm syringe-driven filter (Millipore) into new (acid leached/washed) PP tubes, acidified with 500 µL of ultrapure nitric acid (JT Baker, Ultrex II) and stored refrigerated until analysis for Ca and Sr by Inductively Coupled Plasma-Atomic Emission Spectrometry (ACTIVA M, Horiba Jobin Yvon) and Ba by Dynamic Reaction Cell ICPMS (DRC ICP-MS; Perkin-Elmer). As for otoliths, Sr and Ba were standardised to calcium (i.e. Sr/Ca, Ba/Ca) and converted to weight ratio (mg Sr g^−1^ Ca for Sr/Ca and μg Ba g^−1^ Ca for Ba/Ca), thus giving concentration ratio values.

### Otolith microchemistry analysis

A random subset of otoliths of 62 adults and 70 YOY were analysed with an IR 1 030 nm femto-second laser (Alfamet-Novalase, France) in conjunction with an ICPMS (Inductively Coupled Plasma Mass Spectrometry) Elan DRC II (Perkin-Elmer) for Sr/Ca and Ba/Ca determination. A linear raster scan ablation (width: 30 µm) was taken along the longest radius of the otolith (see Tabouret et al. [37] for details). Concentrations in ^43^Ca, ^86^Sr and ^138^Ba were measured along a transect from the core to the edge of the otolith after a pre-cleaning ablation (50 µm s^−1^). Analytical accuracy was assessed with the fish certified otolith reference material N°22 (National Institute of Environmental Studies, Japan) and three glass reference material N°610, 612, 614 (National Institute of Science and Technology, USA). ^43^Ca was used as an internal standard for each ablation to check for variation in ablation yield. Strontium and barium were standardised to calcium (i.e. Sr/Ca and Ba/Ca) based on the stochiometry of calcium carbonate (389 000 µg Ca g^−1^ otolith) [38], as these elements can substitute for calcium in the otolith matrix [39]. The average detection limit based on three standard deviations (SD) of the blank gas was 20 µg g^−1^ for ^86^Sr/^43^Ca and 0.31 µg g^−1^ for ^138^Ba/^43^Ca.

### Otolith isotopic analysis (carbon and oxygen isotopes)

The stable isotope composition in carbon and oxygen of the larval stage (18 adults and 42 YOY randomly selected from the sample set) and of the juvenile stage (8 adults and 43 YOY randomly selected) was achieved using a microMill (New wave research, ESI, USA) and an isotopic ratio spectrometer (IRMS Finnigan Mat Delta +). Spots of 150 µm were realised for both life stages. To avoid possible contamination by organic matter (<1 wt %), the samples were baked at 380°C for 45 minutes under vacuum [40]. The results are given in the conventional (δ ‰) notation expressed in parts per mil against the V-PDB standard (Vienna Pee Dee Belemnite [41]) where:
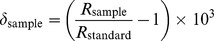



Accuracy was 0.04‰ and 0.05‰ respectively for the carbon and the oxygen isotopes.

### Ageing

Age (in years) of all adult soles was estimated on the otolith transversal plan by scoring the number of annual marks. Distances from the core to each annual mark were measured (μm) for evaluating otolith growth during each life stage. Since individuals in nurseries were less than one year of age, no annual rings were visible. Here, only the distance between the core and the edge of the otolith (μm) was taken as a measure of otolith growth. Distances were measured using a stereomicroscope (Leica MZ) with attached camera (Médiacybernétic™ evolution LS color) and TNPC 4 software (Noesis™-Ifremer).

### Statistical analysis

Otolith radius length was compared by ANOVA with LSD *post-hoc* test to assess differences in otolith growth between nurseries, after testing for normality and homogeneity of variance with Kolmogorov-Smirnov (KS) and Levene tests. Differences between seawater and river water elemental ratios were assessed by Kruskal-Wallis ANOVA (KW) with Mann-Whitney *post-hoc* test.

### Connectivity studies

The estimation of the contribution of one life stage to the next life stage was assessed by two complementary approaches based on the otolith microchemistry: the mixed stock and the individual profile approaches.

#### The mixed stock approach

The contribution of habitats used by larvae and juveniles to adult stocks was determined by comparisons of otoliths of YOY reflecting the larval and juvenile stages to the sections reflecting the same stages in adult otoliths. Figure 3 illustrates which otolith sections of the different life stages were used for this approach. The chemical baselines from each possible juvenile habitat (larval and juvenile) were characterised based on the signatures observed in otoliths of YOY collected in nurseries. Signatures of the otolith subsection of adults corresponding to larval or juvenile life stages were then compared to these baselines by PCA. As suggested by others studies [42], [43], these comparisons by PCA reduced the bias inherent to the fact that not all possible nurseries could be sampled due to logistical constraints. Signatures of individuals that were out of the 95% confidence ellipse performed around the baseline signatures were removed from adult datasets and their proportion was calculated.

The remaining adults constituted the final adult datasets used for the estimation of the relative contributions of larval and/or juvenile habitats. Maximum likelihood estimation (MLE) was achieved to determine the contribution of habitats used by larvae and juveniles to adult populations. Sr/Ca and Ba/Ca ratios were log_10_ transformed to meet normality. Proportions and standard deviations of direct MLE of mixed stocks were obtained with HISEA software [43], [44] using bootstrapping with 1 000 resamples of baseline (YOY signatures) and adult datasets. The direct maximum likelihood estimator is a constrained non-linear maximisation problem that is calculated by maximising the likelihood using the expectation-maximisation algorithm (for more details see Millar [44]).

Analyses focused on Sr/Ca and Ba/Ca ratios, as their simultaneous use in ambient water and otoliths has proven powerful in studies of the environmental migratory history of fish [45], [46]. Their variations in otoliths are influenced by both ambient water composition and physiological processes [38], [46]. Although physiological and temperature-salinity interactions can alter the Sr/Ca chemical composition of otoliths [47], this ratio is usually related to water concentration and in many cases indirectly to salinity (higher ratios in otoliths of marine fish than in brackish or freshwater) [45], [47], [48]. In contrast, the Ba/Ca ratio is generally higher in rivers than in marine water. For the larval stage study, the baseline dataset was constituted by mean Sr/Ca and Ba/Ca ratios measured on the larval life stage of YOY collected in nurseries. High Ba/Ca and low Sr/Ca ratios were usually associated to marine water influenced by terrestrial inputs of the Rhône River, whereas low Ba/Ca and high Sr/Ca ratios were associated to marine water without significant terrestrial influence [49]. For the juvenile stage study, the baseline was constituted by mean Sr/Ca and Ba/Ca ratios of the YOY juvenile stage. Here, high Sr/Ca ratio and low Ba/Ca ratios reflected nurseries with high salinity (e.g., Thau), whereas low Sr/Ca reflected nurseries with lower salinities (e.g., Mauguio and Berre) [49] (Figure S1). Assignment of individuals was done to these general habitats. Because samples in this study were not all from the same time period and signatures can change with time, the reproducibility of patterns over time was tested prior to further analysis (Table S1 in File S1). Fish from Thau showed higher Sr/Ca ratios and Ba/Ca in 2008 than in other years, whereas fish from Mauguio showed high Sr:Ca in 2004. Nevertheless fish from Thau were characterised by consistently and distinctly higher values than fish from Mauguio and Berre. The general chemical properties of nurseries with high salinity and nurseries with low salinity thus remained distinct despite of some degree of inter-annual variability. This conservation of patterns and the correspondence of signatures observed in life stages of YOY and adults (PCA results) suggest that our classification scheme was suitable for the purposes of this study.

#### The individual profile approach

The estimation of the contribution of one stage to the next stage in adult populations required the study of individual profiles. Analyses were assessed on the same individuals used for reclassification by MLE. The Ba/Ca and Sr/Ca ratios of the inner sections of the otoliths of adults corresponding to the larval and juvenile stages (Figure 3) were compared to the corresponding mean signatures recorded in otoliths of YOY collected in the nurseries. The latter do not present inter-annual differences [49] (Table S2 in File S1). The individual chemical profiles of the larval and juvenile stages of adults were compared visually to the average chemical profiles of YOY and classified according to their similarity. Both chemical elements are good indicators of water salinity and of the habitats. However during the larval life stage of common soles, Sr/Ca ratios presented low variations, whereas Ba/Ca ratios showed strong variations. It cannot exclude the possibility that these patterns were influenced by ontogenetic effects, as described by de Pontual et al. [50]. This was the reason why Sr:Ca ratios were not used to determine habitats during the larval life in the individual profile approach, but Ba:Ca ratios. During the juvenile life stage, the opposite was observed with low variations for Ba/Ca ratios and strong variations for Sr/Ca ratio. Due to the lack of variations of the elements according to life stage, only the element which presented variations was used for reclassification. Then, to quantify the proportional contribution of one life stage to the next, a classification scheme was used. Specifically, otoliths were classified as follows: (1) section corresponding to the larval stage with high Ba/Ca ratios >40, larvae mostly influenced by terrestrial inputs; low Ba/Ca<40, larvae mostly influenced by marine waters without significant terrestrial inputs; (2) section corresponding to juvenile stage with low Sr/Ca ratios <5, juvenile influenced by low salinity water; high Sr/Ca ratio >5, juvenile influenced by high salinity water, corresponding to brackish and high salinity nurseries respectively.

#### Stable isotope approach

The stable isotope analysis exploits the principle that otoliths are formed near the isotopic equilibrium between the newly accrued otolith material and the ambient water surrounding a fish, albeit with element specific differences. The δ^13^C reflects principally the dissolved inorganic carbon (DIC) signatures of the waters, and secondly the fish metabolism [51]. In contrast, otolith δ^18^O incorporated in close equilibrium with the δ^18^O of the ambient water, which in term tends to be lower in freshwater than in brackish and marine waters. Secondly δ^18^O shows a small temperature effect (inversely correlate) [52]. In marine water, the δ^18^O varies between −0.30 and 0.05 ‰ [52], whereas freshwater such as the Rhône River presents lower values (−11.6 to −9.8 ‰) [53], [54]. This suggests that fish in our study with high δ^18^O were influenced by water with high salinity and those with low δ^18^O by marine sites under the influence of freshwater. In a previous study [49], low δ^13^C values (<−4.6 ‰) in the inner otolith section corresponding to the larval life stage of YOY were associated to marine water under the influence of terrestrial inputs, whereas higher values (δ^13^C>−4.6 ‰) were associated to marine influence without terrestrial inputs. The study also showed significant isotopic differences between the juvenile life stage of YOY from highly saline and brackish lagoons. To assess the influence undergone by adults during both stages, hierarchical analyses were performed on the isotopic signatures of the larval and juvenile stages of adults and YOY. For larval life, the proportion of adults grouped with YOY influenced by terrestrial inputs (low δ^13^C and δ^18^O) or marine waters (higher δ^13^C and δ^18^O) were calculated. For the juvenile life, the proportion of adults grouped with YOY from high saline lagoon or brackish lagoon were calculated. The clustering algorithm was applied using a Ward method with Euclidian distance.

As sample size (n = 60 *vs* n = 132) and analytical strategies (150 µm spot *vs* continuous profile) differed for isotopic and elemental approaches respectively, the two data set were used independently to infer the connectivity of the sole life stages, which allowed the subsequent comparison of the two estimates.

## Results

### Sr/Ca and Ba/Ca water chemistry

**Figure 4:**
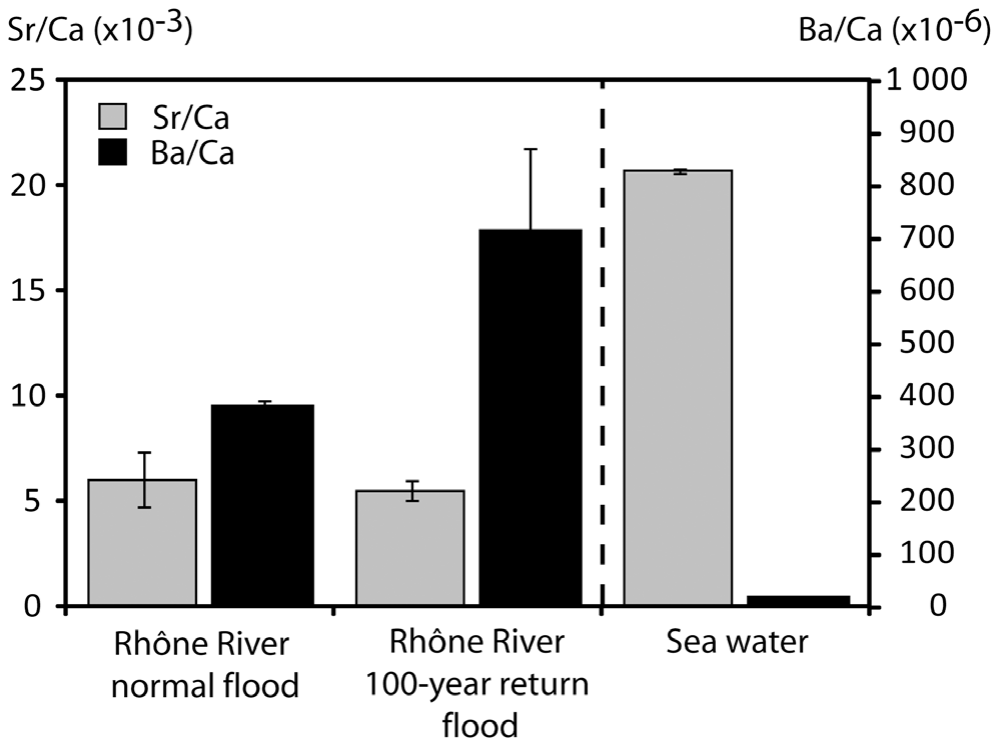
Mean ratios of Sr/Ca (grey) and Ba/Ca (black) of seawater sampled near Marseilles and of the Rhône River sampled during a period of normal discharges (<1 700 m^3^ s^−1^) and a 100-year return flood (>6 000 m^3^ s^−1^) (data from this study and from Ollivier et al. [**36**]).

Large and significant differences in elemental concentrations were observed between seawater and Rhône River water (Figure 4). In particular, the Sr/Ca ratio was 3.7 times higher in seawater (KW χ^2^ = 9.28**), whereas the Ba/Ca ratio was generally higher (KW χ^2^ = 13.75***) and positively correlated with river flow rate in Rhône River water (almost 50 times higher than seawater during the 100-year return flood in December 2003).

### Connectivity studies

#### The mixed stock approach

**Figure 5:**
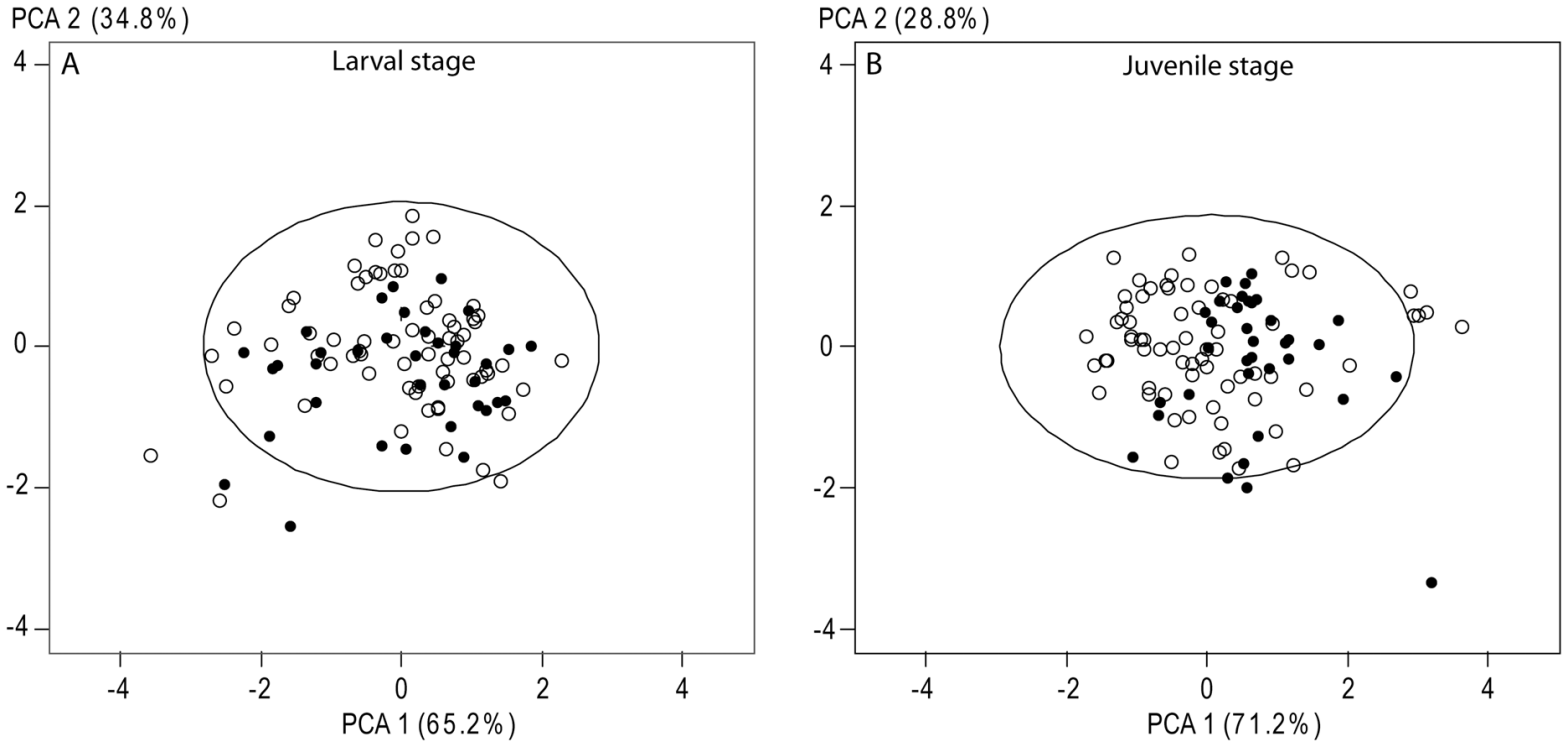
Principal component analysis comparing the microchemistry composition of adults born in 2005–06 (mixed stock group – black spots) and YOY (baseline group – white spots) for the larval (A) and the juvenile (B) stages. Ellipses were the 95% confidence ellipse around the baseline group data.

The signatures of larval and juvenile stages in adult otoliths were mainly distributed in the 95% confidence ellipse achieved with YOY signatures (Figures 5A, B). Individuals falling outside this ellipse may have used habitats not included in the analysis during their larval and/or juvenile stage, and were excluded from the further analysis. For 2005–2006, 2 individuals (5.5% of all fish born in that year) were excluded from both, and for 2004–2005, 2 (7.7%) and 3 (11.5%) individuals were excluded from the further larval and juvenile study, respectively.

The MLE analysis showed that 62.7±20.9% and 56.5±25.2% of fish born in 2005–2006 and 2004–2005, respectively, spent their larval life in marine water influenced by Rhône River inputs, whereas the remainder inhabited marine water without terrestrial influence. Regarding the juvenile stage, the estimated contribution of individuals from high-salinity nurseries to adult stocks was 74.6±10.0% for adults born in 2005–2006 and 54.1±14.9% for those born in 2004–2005, whereas the remainder originated from brackish environments.

#### The individual profile approach

**Figure 6:**
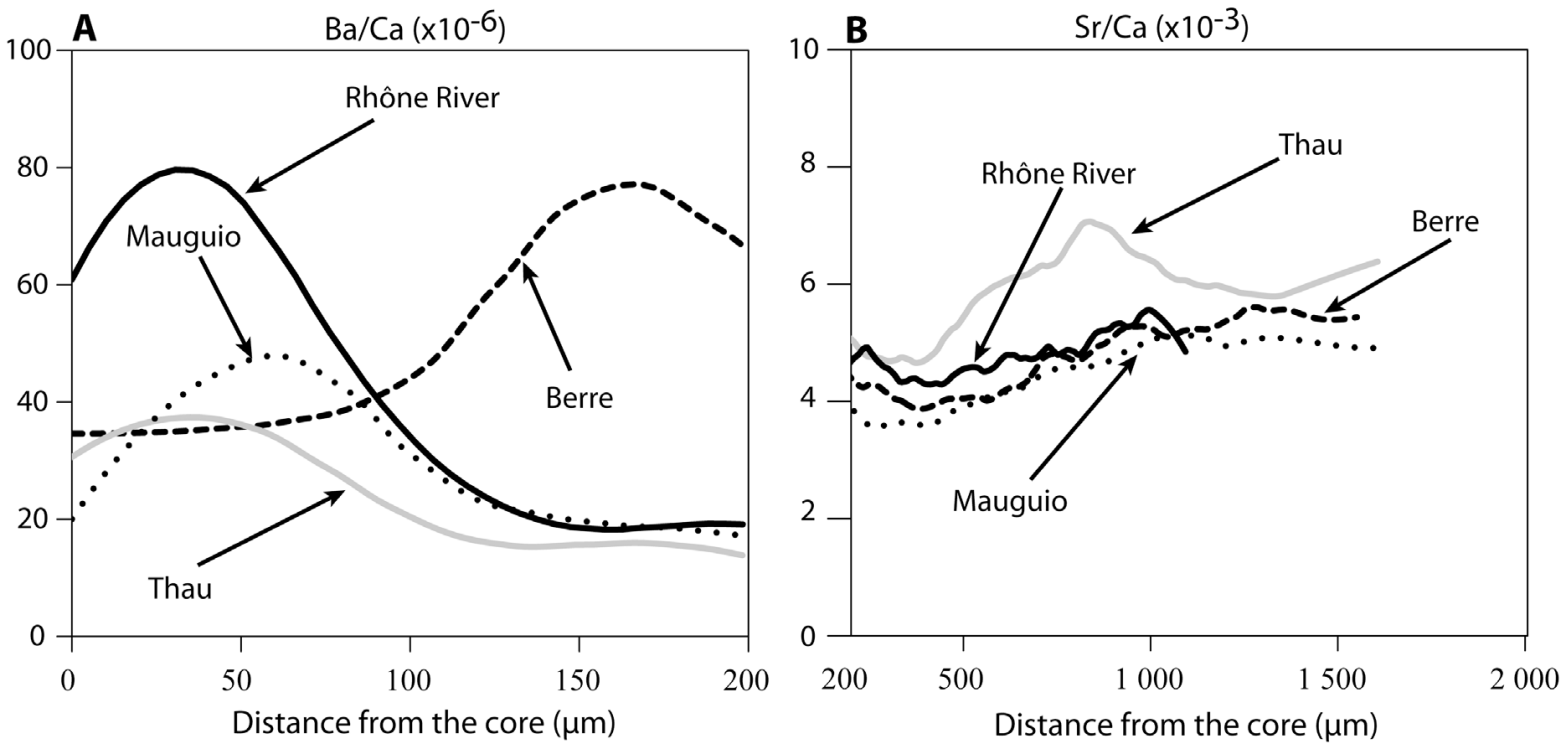
Mean Ba/Ca and Sr/Ca ratios of YOY from nurseries. Ba/Ca ratios are for the larval stage (A) whereas Sr/Ca ratios are for the juvenile stage (B).

Otolith growth rates and Sr/Ca (x10^−3^) and Ba/Ca (x10^−6^) signatures of YOY soles from the different nurseries sampled in this study differed during their larval stage (Figure 6A). In particular, elevated Ba/Ca ratios (up to 80) were observed in the otoliths of YOY collected near the Rhône River mouth and in Berre, compared to values never exceeding 50 for fish captured in the nurseries further away from the river mouth (Thau, Mauguio). At the same time, Berre and Rhône River mouth individuals also presented differences, fish off the Rhône mouth having high ratios at the beginning of the larval stage, contrasting with high ratios at the end of this stage for Berre lagoon. As Ba/Ca ratio were systematically significantly lower in seawater than in the Rhône River water (Figure 4), we assumed that Ba/Ca variations would indicate whether or not the larvae were submitted to the influence of terrestrial inputs from rivers during their marine larval stage. Regarding the YOY in all nurseries, otolith Ba/Ca ratios remained low (∼20) during the entire juvenile stage with negligible variation (Figure S2A). In contrast, while otolith Sr/Ca ratios displayed no difference during the larval life of all YOY (∼4–5) (Figure S2B), they differed during their juvenile stage according to their nursery site, with higher values in high salinity nurseries (>7, e.g., Thau) than in brackish nurseries (<6, e.g., Mauguio, Berre and Rhône River mouth areas) (Figure 6B).

In addition significantly lower otolith growth was recorded in fish collected in the marine coastal nursery near the Rhône River mouth than in those collected in lagoons (Table 2) (KS Z = 0.75, p>0.05; ANOVA F = 22.61, p<0.001). Moreover, the analysis of seasonal means of the daily increment width of otoliths showed that fish from the Rhône River had lower means than fish the brackish nursery Mauguio (Table S3 in File S1). In addition, fish from the Rhône River had a later hatching date (∼15 February on average) than fish collected in brackish nurseries (∼22 January on average), i.e., they were younger at the time of catch (Morat et al. in prep.). The combined differences in increments widths and age could explain the lower distance from the otolith core to the edge compared to fish from Mauguio or Berre. The yet higher distance observed for fish from Thau may be even explained by the earlier hatching period (∼1^st^ December in average) (Morat et al. in prep.). Based on this additional information, the fine-scale distinction between nurseries in this study was possible.

**Table 2:** Number (N) of juveniles caught from the different nurseries in this study, and otoliths radius lengths (μm) (mean ± sd) for these groups.

Nurseries	N	Mean ± sd (min – max)
Thau lagoon	20	1 319±105 (1 174–1 606)
Mauguio lagoon	20	1 283±121 (1 061–1 597)
Rhône River mouth	10	1 012±53 (925–1 093)
Berre lagoon	10	1 362±139 (1 137–1 551)

#### Larval and juvenile otolith Sr/Ca, Ba/Ca and first year otolith length signatures in adult sole

**Figure 7:**
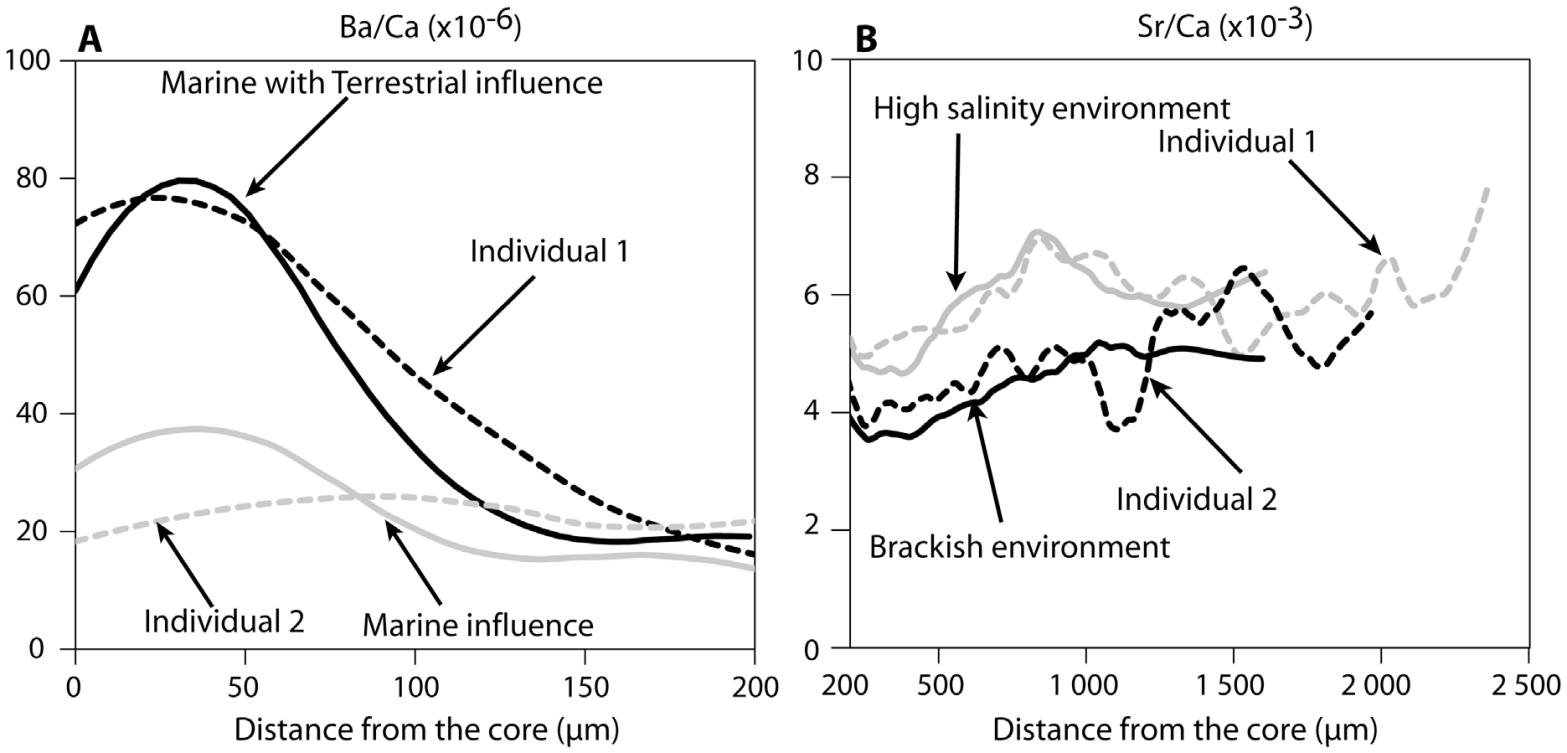
Comparisons of Ba/Ca ratios (A) during the larval stage of adults (dashed lines, individuals 1 and 2) and type individuals captured in nurseries (solid line). Comparisons of Sr/Ca ratios (B) during the juvenile stage of adults (dashed lines, individual 1 and 2) and type individual (average) captured in nurseries (solid line).

**Figure 8:**
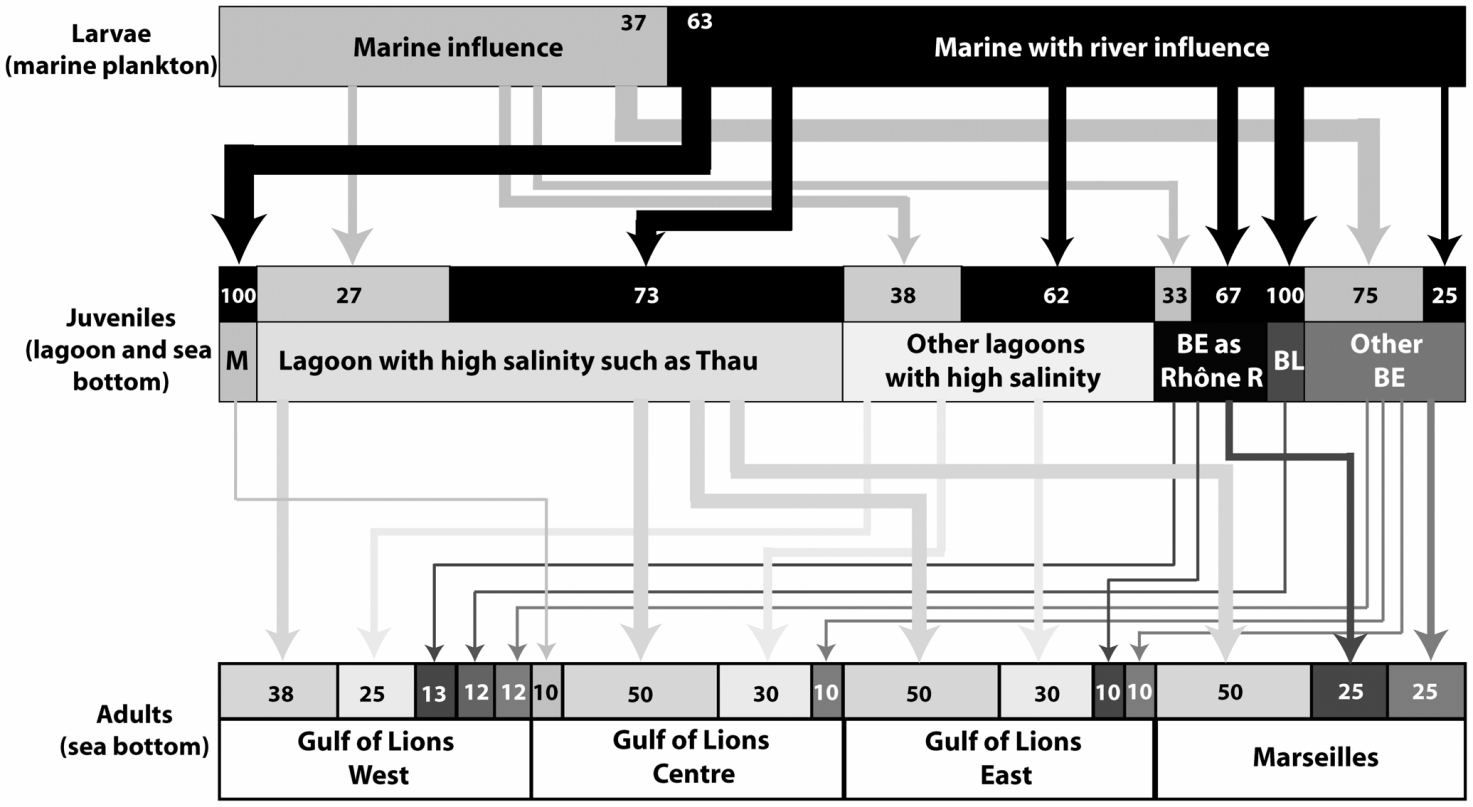
Summary of connectivity between each successive life stages based on Ba/Ca and Sr/Ca ratios of otoliths for the common sole born in winter 2005–2006 and captured in four areas. How to read this figure: the upper part represents the potential habitats used during the larval life stage, the middle part the potential juvenile stage habitats and the lower part the adult stage habitats. Example: in the West of the Gulf of Lions, 38% of adult individuals spent their juvenile stage in lagoons with high salinity such as Thau, 25% from other lagoons with high salinity, etc. The total of each box represents 100%. BE  =  brackish environments, M  =  Marine water.

**Figure 9:**
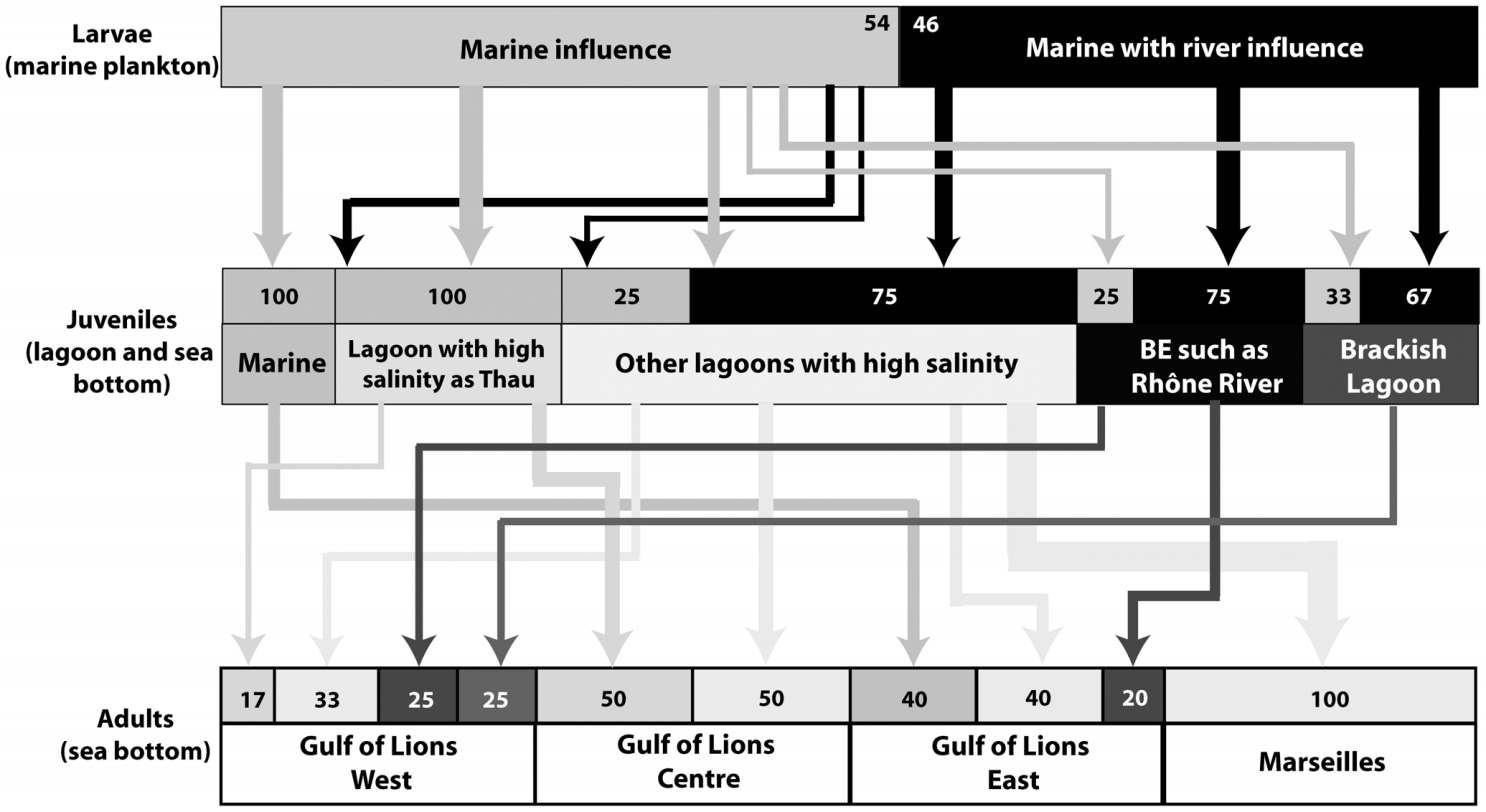
Summary of connectivity between each successive life stages based on Ba/Ca and Sr/Ca ratios of otoliths for the common sole born in winter 2004–2005 and captured in four areas. How to read this figure: the upper part represents the potential habitats used during the larval life stage, the middle part the potential juvenile stage habitats and the lower part the adult stage habitats. **Example**: in the West of the Gulf of Lions, 17%% of adult individuals spent their juvenile stage in lagoons with high salinity such as Thau, 33% from other lagoons with high salinity, etc. The total of each box represents 100%. BE  =  brackish environments.

Based on the comparison of Ba/Ca profiles of the section of adult otoliths corresponding to the larval life to two main patterns identified in the otoliths of YOY (Figure 7A): 63% of adult soles born in 2005–2006 and 46% of those born in 2004–2005 were influenced by river inputs during their larval stage (Figures 8 and 9). These results were similar to those obtained with the mixed stock approach. Based on the elevated otolith Sr/Ca ratios in YOY from the highly saline Thau lagoon compared to the lower otolith Sr/Ca recorded in the moderately saline lagoons (Mauguio, Berre) or river mouth (Rhône), we assume that this ratio reflects the salinity of the environment inhabited by the sole during its juvenile stage. It was thus used here to differentiate two main nursery environments, one with high salinity conditions (high Sr/Ca ratio) and the other with brackish water conditions (low Sr/Ca ratio) (Figure 7B).

Based on the observed variations in Sr/Ca ratio and length of otolith section corresponding to the first year of life of the sole, six potential types of nurseries may be used during this life stage (Figure S3, Table 3): (1) marine water (constant high Sr/Ca ratio), (2) lagoon with high salinity such as Thau (increase in Sr/Ca ratio and high otolith growth during the first year), (3) other lagoons with high salinity (increase in Sr/Ca ratio and low otolith growth), (4) brackish environments such as coastal areas near the Rhône River mouth (low Sr/Ca ratio and low otolith growth), (5) brackish coastal lagoons such as Mauguio and Berre (low Sr/Ca ratio and high otolith growth) and (6) other brackish environments (low but varying Sr/Ca ratio and growth). The cases (3) and (6) were presumably due to coastal lagoons and shallow coastal waters that were not sampled.

**Table 3:** Summary of the unique otolith characteristics corresponding to the six nursery habitats characterised in this study.

Juvenile environments	Sr/Ca	1^st^ year otolith growth	Ba/Ca
**Saline nurseries**			
Marine water	High and constant	Low	Low
Lagoon with high salinity like Thau	High, increasing over time	High	Low
Other lagoon with high salinity	High, increasing over time	Low	Low
**Brackish nurseries**			
Brackish environment like Rhône River mouth	Low	Low	Low
Brackish coastal lagoons (Mauguio and Berre)	Low	High	Low
Other brackish environments	Low with variation	Low with variation	Low

Based on these categories, our results showed that all the possible nursery types contributed to the replenishment of two age-classes of adult sole in this study, but in different proportions according to their geographical location in the GoL (Figures 8 and 9). Most adults born in 2005–2006 (75%, Table 4) potentially spent their juvenile stage in high salinity nurseries, such as Thau lagoon (47%) or other lagoons with high salinity (25%), while only 25% could have lived in brackish water nurseries (Figure 8, Table 4). Similarly, 68% of adults born in 2004–2005 potentially lived in high salinity nurseries and 32% in brackish water nurseries when juveniles (Figure 9). The high contribution of nurseries with high salinity in adult populations was in accordance with the mixed stock approach results. In both years, the proportion of adults with a strictly marine juvenile stage was low (3% in 2005–2006, 9% in 2004–2005). Although the common sole nurseries were similar for both age classes, their contributions to the adult population differed among years. For example, the estimated contribution of nurseries located near the Rhône River to the East GoL adult population was 10% in 2005–2006 and 20% in 2004–2005.

**Table 4:** Synthesis of the estimated contributions of larvae and juveniles from different habitats to replenishment of adult sole populations in different areas of the Gulf of Lions.

	Larvae			Juvenile			Adult	
	2005–06	2004–05		2005–06	2004–05		2005–06	2004–05
Marine influence	37	54	High salinity	25	45	West	3	13
						Centre	13	14
						East	9	13
						Marseilles	0	5
			Brackish environments	12	9	West	3	9
						Centre	0	0
						East	3	0
						Marseilles	6	0
Marine under river influence	63	46	High salinity	50	23	West	12	13
						Centre	16	5
						East	16	5
						Marseilles	6	0
			Brackish environments	13	23	West	7	18
						Centre	3	0
						East	3	5
						Marseilles	0	0

#### Comparison of otolith stable isotope signatures between YOY and adult sole

**Figure 10:**
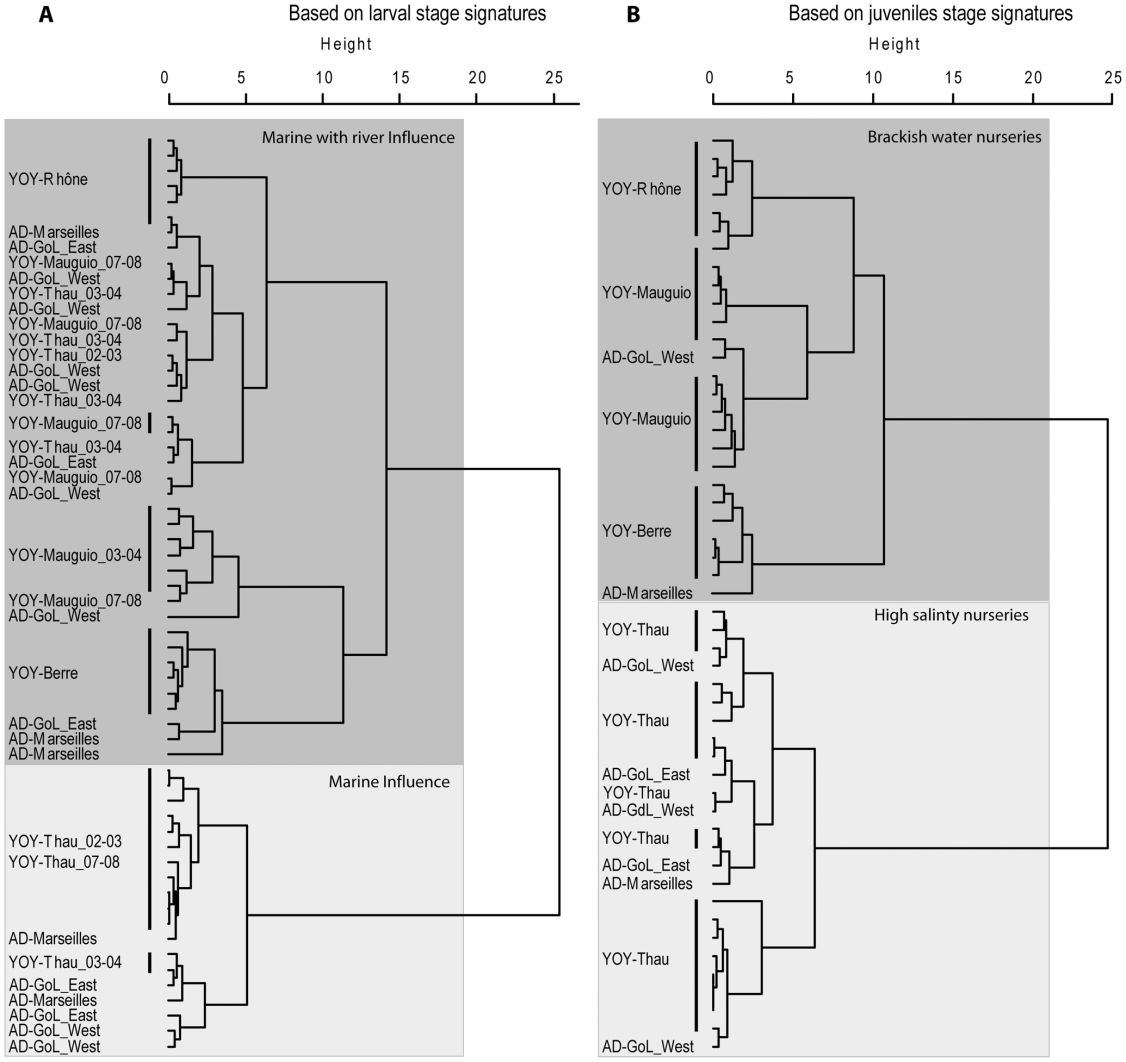
Hierarchical analyses achieved on the isotopic composition (O and C) of the larval stage (A) and juvenile stage (B) of adults born in winter 2004–05 (AD) and YOY. GoL  =  Gulf of Lions.

Contrary to the signatures observed in YOY (Table 5), the signatures of the inner section of otoliths of adults corresponding to the larval and juvenile stages showed a high variability in the 3 sites (GoL West, GoL East and Marseilles, Table 5). This result suggested heterogeneity of origin of these fishes. The hierarchical analysis on the larval stage differentiated two groups of soles based on the isotopic signatures, mainly linked to δ^13^C and secondarily to δ^18^O (Figure 10A). Group one presented fish with low δ^13^C and low δ^18^O (Table 5), indicating a river influence during larval life, and comprised YOY collected in Mauguio, Rhône, Berre and Thau (03–04) as well as adults from all sites. The second group included YOY from Thau (the two other years) and adults from all sites with high δ^13^C and a more positive δ^18^O value (Table 5), indicating a marine influence during the larval stage (Figure 10A). Compared to other nurseries, larvae that have colonised Thau lagoon were characterised by inter-annual variability in water masses influence during their larval stage. It was evidenced that 67% of the adults born in 2004–2005 corresponded to fish subject to imprint of river inputs. Similarly, the juvenile stage signatures evidenced two groups, one corresponding to brackish water (low δ^13^C and δ^18^O) and the other to high salinity nurseries (high δ^13^C and δ^18^O). About 75% of adults spent their juvenile stage on high salinity nurseries (pooled with YOY from Thau), whereas the others (pooled with Mauguio, Berre and Rhône) spent their juvenile stage in brackish waters (Figure 10B, Table 6).

**Table 5:** Stable isotope composition (‰ V-PDB) (mean ± sd) measured in the inner section of otolith of adults (Ad) and young-of-the-year (YOY) corresponding to the larval and juvenile stages.

	Larval life stage			Juvenile life stage		
	N	δ^13^C	δ^18^O	N	δ^13^C	δ^18^O
YOY- Thau 07–08	6	−2.29±0.20	1.54±0.22	6	−2.58±0.21	1.21±0.06
YOY- Thau 03–04	6	−4.95±0.99	0.47±0.18	6	−3.93±0.82	0.72±0.33
YOY- Thau 02–03	6	−2.63±1.48	0.88±0.46	7	−2.89±1.03	0.36±0.51
YOY- Mauguio 07–08	6	−6.49±0.86	0.15±0.35	6	−7.08±0.43	−0.94±0.28
YOY- Mauguio 03–04	6	−8.80±0.50	−0.95±0.54	6	−8.33±1.48	−0.12±0.30
YOY- Berre 07–08	6	−6.08±0.34	−1.62±0.29	6	−6.28±0.49	−2.36±0.32
YOY- Rhône	6	−6.66±0.59	1.84±0.32	6	−6.14±0.65	1.13±0.26
Ad-GoL West	8	−5.62±2.40	0.18±1.33	4	−4.46±2.37	0.62±0.71
Ad-GoL East	5	−4.97±1.42	0.25±1.75	2	−4.08±0.86	0.35±0.70
Ad-Marseilles	5	−4.19±1.47	−0.37±1.91	2	−5.28±1.86	−1.16±3.75

GoL  =  Gulf of Lions. N: number of individuals.

**Table 6:** Seasonal variations in nursery salinity (mean ± sd or range values).

Nurseries	Spring	Summer	Categories	Sources
Berre lagoon	27.4±0.7	28.6±2.2	Brackish	Gipreb unpubl. data
Rhône River mouth	6.0–37.0	6.0–37.0	Brackish	Previmer 2012*
Mauguio lagoon	20.2±3.3	20.0±2.2	Brackish	IFREMER 2009
Thau lagoon	37.4±0.2	38.6±1.2	High salinity	IFREMER 2009
Bages-Sigean lagoon	33.0–36.0	35.0–40.0	High salinity	IFREMER 2009
Leucate lagoon	35.0–38.0	36.0–42.0	High salinity	IFREMER 2009

* http://www.previmer.org/.

## Discussion

Exchanges of offspring between populations (through larval and juvenile dispersion), known as ecological connectivity, are major determinants of the population structures of animals [1]. Consequently, qualitative and quantitative understanding of these exchanges is necessary when assessing the persistence of fish populations, and for the effective management of coastal areas and particular fisheries [10]. To our knowledge, this study was one of the first to combine multi-elemental fingerprints, individual chemical profiles and stable isotope ratios of fish otoliths, as well as analyses of the chemical features of the inhabited water masses from different habitats, in order to characterise the habitats of fish occupied during their different life stages. Our finding of a relevant contribution of all main available nursery types to the replenishment of adult populations of soles in the GoL [29], [55] suggests that these populations were demographically open (exchange of individuals between populations). This would be in accordance with panmixia in the GoL, confirming suggestions of the prior genetic studies based on allozymes [56] and exon-primed intron-crossing [57] in the NW Mediterranean (GoL and Ebre delta).

### Reproducibility of pattern in times

The chemical signatures of otoliths can vary in time. Depending on the study, the observed variability can affect a range from no element to all measured elements (for review see table 4 in Gillanders 2002 [58]). In our study, temporal variation in elemental concentration was observed for Thau (Table S1 in File S1) and Mauguio lagoons (Table S2 in File S1). Nevertheless fish from Thau were characterised by consistently and distinctly higher values of Sr/Ca than fish from Mauguio, i.e., broad patterns persisted despite smaller scale variation. The mismatch in sampling of YOY and adults could increase the variability in the dataset used for the reclassification. However, the PCA performed with YOY and adult signatures showed that only few adults could not be matched to potential sources (11.5% of fish excluded). This confirms the notion that conditions in nursery habitats sampled in this study are relatively homogenous in time.

### Otolith fingerprints as indicators of the water masses inhabited by fish

Characterisation of connectivity patterns between populations or life stages of the common sole has been studied by tagging [59], genetic analysis [56], muscle contaminant signatures [60] and stable isotope composition [28], otolith shape [55] and elemental or stable isotope ratios of otoliths [25], [31], [61]. In our study, elements that present strong relationships with water characteristics have been used successfully for studying fish migration and ecological connectivity, as observed in others studies [45], [46]. The highly elevated Ba/Ca ratios observed in the Rhône River water compared to marine water in this study fit our expectations, since river water is generally characterised by higher Ba/Ca ratios than marine water [45], [47], [48], and since the Rhône represents the most important Ba provider to the GoL. In this study, the high proportion of soles that displayed both high otolith Sr/Ca ratios (indicative of marine waters) and high Ba/Ca ratio (indicative of freshwater influence) during the larval stage was unexpected. However, recent experiments showed that riverine transported particulate materials can alter the barium concentration of seawater without changing the Sr and Ca concentrations by means of desorption mechanism and formation of secondary phases [62]. Taking into account the total amount of suspended matter entering the GoL during Rhône River floods [36] and its wide dispersion, it is likely that this mechanism can lead to the occurrence of enriched zones in Ba/Ca ratio without significantly decreasing the Sr/Ca. Similarly, the δ^13^C ratio of otoliths reflect principally the dissolved inorganic carbon (DIC) signatures of the waters (secondarily the fish metabolism), and the δ^18^O ratio of otoliths is dependent on the δ^18^O values of the water, with a small inverse temperature effect [52]. In marine water, the δ^13^C and δ^18^O varied between 0.0 and 2.0 ‰ and between −0.30 and 0.05 ‰ respectively [52], [53]. In the Rhône River water, they have lower values between −10.2 to −9.0 ‰ for δ^13^C and −11.6 to −9.8 ‰ for δ^18^O [51], [53]. Thus, in this study, fishes with high Ba/Ca ratio (>50), low δ^13^C (−5.0/−10.0 ‰) and low δ^18^O in otoliths during the marine larval stage were likely associated with Rhône River inputs (dissolved and suspended particulate) in the surface water layer of the GoL. In contrast, fish with low Ba/Ca ratio (<50) and higher δ^13^C values (−3.5/−2.0 ‰) and δ^18^O were likely associated to influence by marine water without significant terrestrial inputs.

Otolith Sr/Ca variations in fish are likely to be influenced by both ambient water composition and physiological processes [38], [63]. However Sr/Ca ratio is usually related to water concentration and in many cases indirectly to salinity. Our life-history reconstructions are mainly based on several year average of Sr/Ca ratio from otoliths of YOY collected in nurseries with contrasting biogeochemical characteristics. It allowed us to relate the elevated proportion of fish with high Sr/Ca ratios in otolith to highly saline nurseries, and low proportions of fish with lower ratios to brackish nurseries (Table 4). Within these two different saline-type nurseries, average otolith growth rate of the first year of life allowed differentiating nurseries having similar saline-type characteristics, but probably different food resources.

### Connectivity in common sole adult populations

Our results evidenced that all nursery types are colonised by larvae independently of the prior influence undergone, suggesting an intermixing of larval pools when they settled in nurseries. Moreover all potential nursery types contributed to the replenishment of adult sole populations in the GoL, whatever their location. However, the adults of the common sole appear to be rather sedentary and do not undertake wide migrations between populations [60], [64]. Indeed, distinctly different profiles of long-lived contaminants (PCBs, pesticides and heavy metals) in common soles from four regions across the GoL indicate a clear spatial differentiation [60] that indicated that little adult migration occurred between these areas. Our study thus suggests that the high connectivity of common sole populations in the GoL observed in previous studies (genetic study) is achieved during the early life stages larvae and juveniles in two successive steps.

During the larval life stage, common sole are mainly transported by currents as passive particles during a few days, but present quickly oriented swimming movements that play a role on their dispersion and migration towards suitable coastal nursery grounds [65]. In our study, the high dispersion of sole larvae around the GoL and the high “self-recruitment” of larvae influenced by the Rhône River to nurseries near the Rhône River (67–75% of fish in both age-classes of adult sole, Figures 8 and 9) can be explained by these parameters (passive transport and swimming activity). During their 20 to 46 day pelagic larval stage [66], common sole larvae can disperse over long distances in the Irish Sea and Bristol channel (>100 km, [64]). In the GoL, water circulation is driven by the North Current flowing from East to West and counter-currents directed to the coast [67]. Waters from the Eastern part the GoL, submitted to Rhône River inputs, are deflected towards the Western part of the gulf by the North Current and should drive sole larvae influenced by the Rhône River to this region. In the middle and the Eastern part of the GoL, counter-currents can drive offshore waters and the associated fish larvae to the coast near Thau and Mauguio lagoons (Centre) and near the Rhône River (East). Moreover, the swimming activity of common sole larvae (5–40 cm min^−1^ [68]), joined to nycthemeral vertical migrations, can concomitantly act with currents for oriented transport [65], [66]. Larval choice of nursery habitats presenting the higher food availability could be influenced by physical and chemical factors like flavour, smell, temperature or salinity of nursery [69], as well as food availability [70] and chemical components of preys [71].

Common sole juveniles migrate to recruit to adult populations after a few months spent in nurseries. Like other flatfish species, they present high swimming ability [72]. In our study, potential sole nurseries had a maximum distance of 150 km from adult habitats. The first migration distances in the Mediterranean fall into the range of migration abilities described for juveniles assessed by tagging in the Bay of Biscay (40–80 km) [59] and by otolith microchemistry of three flatfish species, including the common sole, from the Portuguese coast (>250 km, [31]). This high migration ability of soles at the juvenile stage was probably due to an elevate cruising speed as observed for plaice and common dab juveniles (80–100 cm s^−1^ and 26 cm s^−1^ respectively [72]).

In the GoL, each local adult population was comprised of juveniles that originated from most, if not all, nursery types, suggesting intermixing of juvenile populations in the process of recruiting to deeper adult habitats. In addition, variations in patterns found between 2005 and 2006 (Figures 8 and 9, Table 4) evidenced fluctuations over time in the connectivity processes, thus suggesting that the relative importance of the main driving factors may change even at a short temporal scale [42], [73]. The inter-annual differences in this study could reflect changes in the inputs of the Rhône River during the larval life of soles, although no differences in Rhône River flow rate were observed in winter and in spring for the two years (Compagnie nationale du Rhône, pers. com.). Alternatively, differences could also reflect either change in the physico-chemical conditions of nurseries used by fish during the juvenile stage and/or changes in fishing pressure in these habitats between both years. This latter hypothesis could contribute to the selection of individuals from one or the other type of nurseries in adult populations of the GoL.

Common sole nurseries are often described as brackish estuarine environments submitted to organic matter enrichment by river inputs [27], [74], [75]. On the opposite, our study highlighted the importance of nurseries with high salinity (nurseries for 68–72% of soles, Table 4), such as Thau lagoon, for the common sole in the GoL in addition to these brackish environments. The rivers around the GoL present no large estuaries like Atlantic rivers (Seine, Gironde, Loire, Tagus, Thames, etc.), while the larger, the Rhône River, is characterised by delta. Lagoons around the GoL with their high productivity could have the same role as nurseries along French Atlantic coastline. Other coastal lagoons, such as Leucate and Bages-Sigean (see Figure 2) are known to also serve as nurseries for the common sole in the GoL and most likely contribute to the replenishment of adult populations. They presented the same range of water salinity in spring and summer than Thau lagoon (Table 6), and could thus contribute to fish that have been classified as spending their juvenile life in lagoons with high salinity. However, as the otolith growth during the first year of life in these lagoons was not known, they could not be identified in adult profiles. Similarly several small coastal rivers are located around the GoL and could contribute to the fish category including individuals that have been classified as spending their juvenile life in brackish environments.

Mortality during the early life history stages of marine fish is high due to strong selection [76]. The “bigger is better hypothesis” suggests that vulnerable stage duration is reduced for fast-growing individuals [77], [78]. These individuals are less vulnerable to predation than slow-growing individuals. We found higher growth rates in coastal lagoons rather than in coastal nurseries near the Rhône River mouth. Among these lagoons, the brackish lagoons (Mauguio and Berre) present better growth conditions (low salinity, high temperature) [79]. However their contribution to adult populations of the GoL was low for both years studied. This result appear to contradict the “bigger is better hypothesis”, however, brackish lagoons are subjected to high fishing pressure from small scale artisanal fisheries. The fast-growing individuals escape predation but they reach more quickly a fishing size for small scale fisheries (∼20 cm in French Mediterranean Sea). Our results suggest that bigger is not always better, especially in the context of high fishery pressure.

The patterns in ecological connectivity of the common sole in the GoL observed here underscored the major role of coastal lagoons as nursery habitats in this area. They can be described as essential fish habitats, as they highly contributed to the renewal of adult fish populations. Many other commercial fish species like flatfish (*S. senegalensis, Platichthys flesus*, etc.), seabream (*Sparus aurata*), seabass (*Dicentrarchus labrax*), eel (*Anguilla anguilla*) or anchovy (*Engraulis encrasicolus*) also use coastal lagoons as nurseries during their juvenile stage. The importance of these habitats in the life cycle of these fishes should be studied for a better understanding of the ecological connectivity and the replenishment of exploited fish population.

## Supporting Information

Figure S1 **Canonical discriminant analysis performed with Sr:Ca and Ba:Ca of fish collected in nurseries in order to evaluate the difference between nurseries (in 2008).** Function 1 and 2 are linear combinations of descriptors that maximize the Wilks λ. Each function represents a part of the total variability (in %) of dataset. The Wilks λ allows assessment of the performance of the discriminant analysis. The values of λ range from 0 to 1, and the closer the λ is to 0, the better is the discriminating power of the CDA.
(TIF)

Figure S2 **Mean Ba/Ca and Sr/Ca ratios of YOY from nurseries. Ba/Ca ratios are for the juvenile stage (A) whereas Sr/Ca ratios are for the larval stage (B).**
(TIF)

Figure S3 **Example of comparison between individuals from the 6 potentials nursery types inhabited by soles during the juveniles stage (solid lines) and type curve of nurseries with high salinity (grey dashed line) and brackish environments (dark dashed line).** The arrows represent the annual marks observed in adult profiles. Common soles leave nurseries at the end of the first year of life, thus the comparisons between potentials types of nurseries and adults profiles were performed only for this period.
(TIF)

File S1 **Includes Tables S1 - S3.** Table S1. Results of MANOVAs performed with Sr:Ca and Ba:Ca ratios of fish collected in nurseries in order to evaluate the variations over time and the differences between nurseries. Table S2. Mean (±sd) and results of t-test performed on the Sr:Ca and Ba:Ca measured on the larval and juvenile life stages of YOY from Mauguio in 2004 and 2008. Table S3. Seasonal mean (±sd) of the daily increment width of otoliths from YOY collected in nurseries and results of comparisons between nurseries (ANOVAs and Fischer LSD).
(DOC)

## References

[1] Cowen RK, Paris CB, Srinivasan A (2006) Scaling of connectivity in marine populations. Science 311: 522–527. 16357224 10.1126/science.1122039

[2] Jones GP, Milicich MJ, Emslie MJ, Lunow C (1999) Self-recruitment in a coral reef fish population. Nature 402: 802–804.

[3] Frisk MG, Jordaan A, Miller TJ (2013) Moving beyond the current paradigm in marine population connectivity: are adults the missing link? Fish Fish: doi: 10.1111/faf.12014.

[4] Lagardère F, Amara R, Joassard L (1999) Vertical distribution and feeding activity of metamorphosing sole, *Solea solea*, before immigration to the Bay of Vilaine nursery (northern Bay of Biscay, France). Environ Biol Fishes 56: 213–228.

[5] Leis JM (2006) Are larvae of demersal fishes plankton or nekton. Adv Mar Biol 51: 59–141. 10.1016/S0065-2881(06)51002-8 16905426

[6] Lecchini D, Planes S, Galzin R (2005) Experimental assessment of sensory modalities of coral-reef fish larvae in the recognition of their settlement habitat. Beh Ecol Sociobiol 58: 18–26.

[7] Almany GR, Berumen ML, Thorrold SR, Planes S, Jones GP (2007) Local replenishment of coral reef fish populations in a marine reserve. Science 316: 742–744. 17478720 10.1126/science.1140597

[8] Cowen RK, Lwiza KMM, Sponaugle S, Paris CB, Olson DB (2000) Connectivity of marine populations: open or closed? Science 287: 857–859. 10657300 10.1126/science.287.5454.857

[9] Secor DH (1999) Specifying divergent migrations in the concept of stock: the contingent hypothesis. Fish Res 43: 13–34.

[10] Sale PF, Van Lavieren H, Ablan Lagman MC, Atema J, Butler M, et al.. (2010) Preserving reef connectivity: a handbook for marine protected area managers. Currie communications, Melbourne, Australia, May 2010. Coral reef targeted research and capacity building for management program, 2010. 80 p.

[11] Harrison HB, Williamson DH, Evans RD, Almany GR, Thorrold SR, et al (2012) Larval export from marine reserves and the recruitment benefit for fish and fisheries. Curr Biol 22: 1023–1028. 22633811 10.1016/j.cub.2012.04.008

[12] Pauly D, Christensen V, Guénette S, Pitcher TJ, Sumaila UR, et al (2002) Towards sustainability in world fisheries. Nature 418: 689–695. 12167876 10.1038/nature01017

[13] Planes S, Jones GP, Thorrold SR (2009) Larval dispersal connects fish populations in a network of marine protected areas. Proc Natl Acad Sci U S A 106: 5693–5697. 19307588 10.1073/pnas.0808007106 PMC2659712

[14] Mellon-Duval C, de Pontual H, Métral L, Quemener L (2010) Growth of European hake (*Merluccius merluccius*) in the Gulf of Lions based on conventional tagging. ICES J Mar Sci 67: 62–70.

[15] Hobson KA (2007) Chapter 6: Isotopic tracking of migrant wildlife. In: Michener R, Lajtha K, editors. Stable isotopes in ecology and environmental science Second Edition: Blackwell.pp. 155–175.

[16] Hamer PA, Jenkins GP, Coutin P (2006) Barium variation in *Pagrus auratus* (Sparidae) otoliths: A potential indicator of migration between an embayment and ocean waters in south-eastern Australia. Estuar, Coast Shell Sci 68: 686–702.

[17] Patterson HM, Swearer SE (2007) Long-distance dispersal and local retention of larvae as mechanisms of recruitment in an island population of a coral reef fish. Austral Ecology 32: 122–130.

[18] Popper AN, Combs S (1980) Auditory mechanisms in teleost fishes. Am Sci 68: 429–440.

[19] Campana SE, Casselman JL (1993) Stock discrimination using otolith shape analysis. Can J Fish Aquat Sci 50: 1062–1083.

[20] Campana SE, Neilson JD (1985) Mircrostructure of fish otoliths. Can J Fish Aquat Sci 42: 1014–1031.

[21] Fowler AJ, Campana SE, Jones CM, Thorrold SR (1995) Experimental assessment of the effect of temperature and salinity on elemental composition of otoliths using solution-based ICPMS. Can J Fish Aquat Sci 52: 1421–1430.

[22] Borelli G, Mayer-Gostan N, de Pontual H, Boeuf G, Payan P (2001) Biochemical relationships between endolymph and otolith matrix in the trout (*Oncorhynchus mykiss*) and turbot (*Psetta maxima*). Calcif Tissue Int 69: 356–364. 11800233 10.1007/s00223-001-2016-8

[23] Farrell J, Campana SE (1996) Regulation of calcium and strontium deposition on the Otoliths of Juvenile Tilapia, *Oreochromis niloticus* . Comp Biochem Physiol 115A: 103–109.

[24] Gillanders BM, Kingsford MJ (2000) Elemental fingerprints of otoliths of fish may distinguish estuarine ‘nursery’ habitats. Mar Ecol Prog Ser 201: 276–286.

[25] Tanner SE, Reis-Santos P, Vasconcelos RP, França S, Thorrold SR, et al (2012) Otolith geochemistry discriminates among estuarine nursery areas of *Solea solea* and *S. senegalensis* over time. Mar Ecol Prog Ser 452: 193–203.

[26] Demaneche S, Merrien C, Berthou P, Lespagnol P (2009) Rapport R3, Méditerranée continentale, échantillonnage des marées au débarquement. Méthode d'élévation et évaluation des captures et de l'effort de pêche des flottilles de la façade Méditerranée continentale sur la période 2007–2008. IFREMER, France. 217 p.

[27] Salen-Picard C, Darnaude A, Arlhac D, Harmelin-Vivien ML (2002) Fluctuations of macrobenthic populations: a link between climate-driven river run-off and sole fishery yields in the Gulf of Lions. Oecologia 133: 380–388. 28466212 10.1007/s00442-002-1032-3

[28] Dierking J, Morat F, Letourneur Y, Harmelin-Vivien M (2012) Fingerprints of lagoonal life: Migration of the marine flatfish *Solea solea* assessed by stable isotopes and otolith microchemistry. Estuar, Coast Shelf Sci 104–105: 23–32.

[29] Gaertner JC, Chessel D, Bertrand J (1998) Stability of spatial structures of demersal assemblages: a multiple approach. Aquat Living Resour 11: 75–85.

[30] Letourneur Y, Darnaude A, Salen-Picard C, Harmelin-Vivien ML (2001) Spatial and temporal variations of fish assemblages in a shallow Mediterranean softbottom area (Gulf of Fos, France). Oceanol Acta 24: 273–285.

[31] Vasconcelos RP, Reis-Santos P, Tanner S, Maia A, Latkoczy C, et al (2008) Evidence of estuarine nursery origin of five coastal fish species along the Portuguese coast through otolith elemental fingerprints. Estuar Coast Shelf Sci 79: 317–327.

[32] Lagardère F, Troadec H (1997) Age estimation in common sole *Solea solea* larvae: validation of daily increments and evaluation of pattern recognition technique. Mar Ecol Prog Ser 155: 223–267.

[33] Estournel C, Marsaleix P, Auclair F, Ulses C, Herrmann M (2009) Chapitre 16 : Le golfe du Lion, poumon de la circulation méditerranéenne. In: Quae, editor. Le golfe du Lion : Un observatoire de l'environnement en Méditerranée.Versailles, France, pp 257–267.

[34] Lochet F, Leveau M (1990) Transfers between a eutrophic ecosystem, the river Rhône, and an oligotrophic ecosystem, the north-western Mediterranean Sea. Hydrobiologia 207: 95–103.

[35] Miralles J, Arnaud M, Radakovitch O, Marion C, Cagnat X (2006) Radionuclide deposition in the Rhône River Prodelta (NW Mediterranean sea) in response to the December 2003 extreme flood. Mar Geol 234: 179–189.

[36] Ollivier P, Radakovitch O, Hamelin B (2011) Major and trace element partition and fluxes in the Rhône River. Chem Geol 285: 15–31.

[37] Tabouret H, Bareille G, Claverie F, Pécheyran C, Donard OFX (2010) Simultaneous use of strontium : calcium and barium : calcium ratios in otoliths as markers of habitat: Application to the European eel (*Anguilla anguilla*) in the Adour basin, South West France. Mar Environ Res 70: 35–45. 20338633 10.1016/j.marenvres.2010.02.006

[38] Brown RJ, Severin KP (2009) Otolith chemistry analyses indicate that water Sr:Ca is the primary factor influencing otolith Sr:Ca for freshwater and diadromous fish but not for marine fish. Can J Fish Aquat Sci 66: 1790–1808.

[39] Campana SE (1999) Chemistry and composition of fish otoliths: pathways, mechanisms and applications. Mar Ecol Prog Ser 188: 263–297.

[40] Blamart D, Escoubeyrou K, Juillet-Leclerc A, Ouahdi R, Lecomte-Finiger R (2002) Composition isotopique δ18O-δ13C des otolithes des populations de poissons récifaux de Taiaro (Tuamotu, Polynésie française) : implications isotopiques et biologiques. C R Biol 325: 99–106. 11980181 10.1016/s1631-0691(02)01419-1

[41] Coplen TB, Kendall C, Hopple J (1983) Comparison of stable isotope reference samples. Nature 302: 236–238.

[42] Chittaro P, Finley R, Levin P (2009) Spatial and temporal patterns in the contribution of fish from their nursery habitats. Oecologia 160: 49–61. 19214587 10.1007/s00442-009-1282-4

[43] Tanner SE, Reis-Santos P, Vasconcelos RP, Thorrold SR, Cabral HN (2013) Population connectivity of *Solea solea* and *Solea senegalensis* over time. J Sea Res 76: 82–88.

[44] Millar RB (1990) A versatile computer program for mixed stock fishery composition estimation. Canadian Technical Report of Fisheries and Aquatic Sciences 1753: , 1–29.

[45] Elsdon TS, Gillanders BM (2005) Consistency of patterns between laboratory experiments and field collected fish in otolith chemistry: an example and applications for salinity reconstructions. Mar Freshw Res 56: 609–617.

[46] Walther BD, Thorrold SR (2006) Water, not food, contributes the majority of strontium and barium deposited in the otoliths of a marine fish. Mar Ecol Prog Ser 311: 125–130.

[47] Webb SD, Woodcock SH, Gillanders BM (2012) Sources of otolith barium and strontium in estuarine fish and the influence of salinity and temperature. Mar Ecol Prog Ser 453: 189–199.

[48] Bath GE, Thorrold SR, Jones CM, Campana SE, McLaren JW, et al (2000) Strontium and barium uptake in aragonitic otoliths of marine fish. Geochim Cosmochim Acta 64: 1705–1714.

[49] Morat F, Lecomte-Finiger R, Blamart D, Robert M, Letourneur Y (2012) Preliminary indication of ontogenetic and spatial variations in the whole otolith isotopic and elemental signatures of *Solea solea* in the Gulf of Lions (NW Mediterranean). Sci Mar 76: 647–657.

[50] de Pontual H, Lagardère F, Amara R, Bohn M, Ogor A (2003) Influence of ontogenetic and environmental changes in the otolith microchemistry of juvenile sole (*Solea solea*). J Sea Res 50: 199–210.

[51] Solomon CT, Weber PK, Cech Jr JJ, Ingram BL, Conrad ME, et al (2006) Experimental determination of the sources of otolith carbon and associated isotopic fractionation. Can J Fish Aquat Sci 63: 79–89.

[52] Panfili J, de Pontual H, Troadec H, Wright PJ (2002) Manuel de sclérochronologie des poissons. Coédition Ifremer-IRD, Panfili J, de Pontual H, Troadec H, Wright PJ (eds), France, 464 pp. 464 p.

[53] Aucour A-M, Sheppard SMF, Savoye R (2003) d13C of fluvial mollusk shells (Rhône River): A proxy for dissolved inorganic carbon? Limnol Oceanogr 48: 2186–2193.

[54] Celle-Jeanton H, Gonfiantini R, Travi Y, Sol B (2004) Oxygen-18 variations of rainwater during precipitation: application of the Rayleigh model to selected rainfalls in Southern France. J Hydrol 289: 165–177.

[55] Mérigot B, Letourneur Y, Lecomte-Finiger R (2007) Characterization of local populations of the common sole *Solea solea* (Pisces, Soleidae) in the NW Mediterranean through otolith morphometrics and shape analysis. Mar Biol 151: 997–1008.

[56] Kotoulas G, Bonhomme F, Borsa P (1995) Genetic structure of the common sole *Solea vulgaris* at different geographic scales. Mar Biol 122: 361–375.

[57] Rolland J-L, Bonhomme F, Lagardére F, Hassan M, Guinand B (2007) Population structure of the common sole (*Solea solea*) in the Northeastern Atlantic and the Mediterranean Sea: revisiting the divide with EPIC markers. Mar Biol 151: 327–341.

[58] Gillanders BM (2002) Temporal and spatial variability in elemental composition of otoliths: implications for determining stock identity and connectivity of populations. Can J Fish Aquat Sci 59: 669–679.

[59] Koutsikopoulos C, Dorel D, Desaunay Y (1995) Movement of sole (*Solea solea*) in the Bay of Biscay: coastal environment and spawning migration. J Mar Biol Assoc UK 75: 109–126.

[60] Dierking J, Wafo E, Schembri T, Lagadec V, Nicolas C, et al (2009) Spatial patterns in PCBs, pesticides, mercury and cadmium in the common sole in the NW Mediterranean Sea, and a novel use of contaminants as biomarkers. Mar Pollut Bull 58: 1605–1614. 19692097 10.1016/j.marpolbul.2009.07.008

[61] Cuveliers E, Larmuseau M, Hellemans B, Verherstraeten S, Volckaert F, et al (2012) Multi-marker estimate of genetic connectivity of sole (*Solea solea*) in the North-East Atlantic Ocean. Mar Biol 159: 1239–1253.

[62] Jones MT, Pearce CR, Oelkers EH (2012) An experimental study of the interaction of basaltic riverine particulate material and seawater. Geochimi Cosmochim Acta 77: 108–120.

[63] Secor DH, Rooker JR (2000) Is otolith strontium a useful scalar of life cycles in estuarine fishes? Fish Res 46: 359–371.

[64] Symonds DJ, Rogers SI (1995) The influence of spawning and nursery grounds on the distribution of sole *Solea solea* (L.) in the Irish Sea, Bristol Channel and adjacent areas. J Exp Mar Biol Ecol 190: 243–261.

[65] Champalbert G, Macquart-Moulin C, Howell B (1992) Effects of sediment on the settlement of larvae and juvenile sole (*Solea solea* (L.)) in laboratory conditions. Mar Behav Physiol 21: 255–276.

[66] Amara R, Lagardère F, Desaunay Y, Marchand J (2000) Metamorphosis and estuarine colonisation in the common sole, *Solea solea* (L.): implications for recruitment regulation. Oceanol Acta 23: 469–484.

[67] Millot C (1979) Wind induced upwellings in the Gulf of Lions. Oceanol Acta 2: 261–274.

[68] Hunter JR (1980) The feeding behavior and ecology of marine fish larvae. p. 287–330. In: Bardach JE, Magnuson JJ, May RC, Reinhart JM, editors. Fish behavior and its use in the capture and culture of fishes ICLARM Conference proceedings International center for living aquatic resources managements. Manila, Philippines.pp. 512.

[69] Miller J (1988) Physical processes and the mechanims of coastal migrations of immature marine fishes. American Society Symposium 3: 68–76.

[70] Macquart-Moulin C, Champalbert G, Howell BR, Patriti G, Ranaivoson C (1991) La relation alimentataire - fixation benthique chez les jeunes soles *Solea solea* L. métamorphosées. Evidences expérimentales. J Exp Mar Biol Ecol 153: 195–205.

[71] Konosu S, Hayashi T (1975) Determination of a-alanine betaine and glycine betaine in some marine invertebrates. Bulletin of the Japanese Society of Scientific Fisheries 41: 743–746.

[72] Kearn GC (1974) The effects of fish skin mucus on hatching in the monogenean parasite *Entobdella soleae* from the skin of the common sole, *Solea solea* . Parasitology 68: 173–188. 4857040

[73] Sale PF (2004) Connectivity, recruitment variation, and the structure of reef fish communities. Integr Comp Biol 44: 390–399. 21676724 10.1093/icb/44.5.390

[74] Le Pape O, Chauvet F, Désaunay Y, Guérault D (2003) Relationship between interannual variations of the river plume and the extent of nursery grounds for the common sole (*Solea solea*, L.) in Vilaine Bay. Effects on recruitment variability. J Sea Res 50: 177–185.

[75] Vinagre C, Salgado J, Cabral HN, Costa MJ (2011) Food web structure and habitat connectivity in fish estuarine nurseries - Impact of river flow. Estuaries Coasts 34: 663–674.

[76] Bailey K, Houde E (1989) Predation on eggs and larvae of marine fishes and the recruitment problem. Adv Mar Biol 25: 1–83.

[77] Miller TJ, Crowder LB, Rice JA, Marschall EA (1988) Larval size and recruitment mechanisms in fishes: toward a conceptual framework. Can J Fish Aquat Sci 45: 1657–1670.

[78] Meekan M, Vigliola L, Hansen A, Doherty P, Halford A, et al (2006) Bigger is better: size-selective mortality throughout the life history of a fast-growing clupeid, *Spratelloides gracilis* . Mar Ecol Prog Ser 317: 237.

[79] Imsland AK, Foss A, Conceição LEC, Dinis MT, Delbare D, et al (2003) A review of the culture potential of *Solea solea* and *S. senegalensis* . Rev Fish Biol Fish 13: 379–408.

