# Supplementary figures and images for: The Great Melting Pot. Common Sole Population Connectivity Assessed by Otolith and Water Fingerprints
Source: PLoS One. 2014 Jan 27;9(1):e86585. doi: 10.1371/journal.pone.0086585 (PMC3903582; doi:10.1371/journal.pone.0086585)

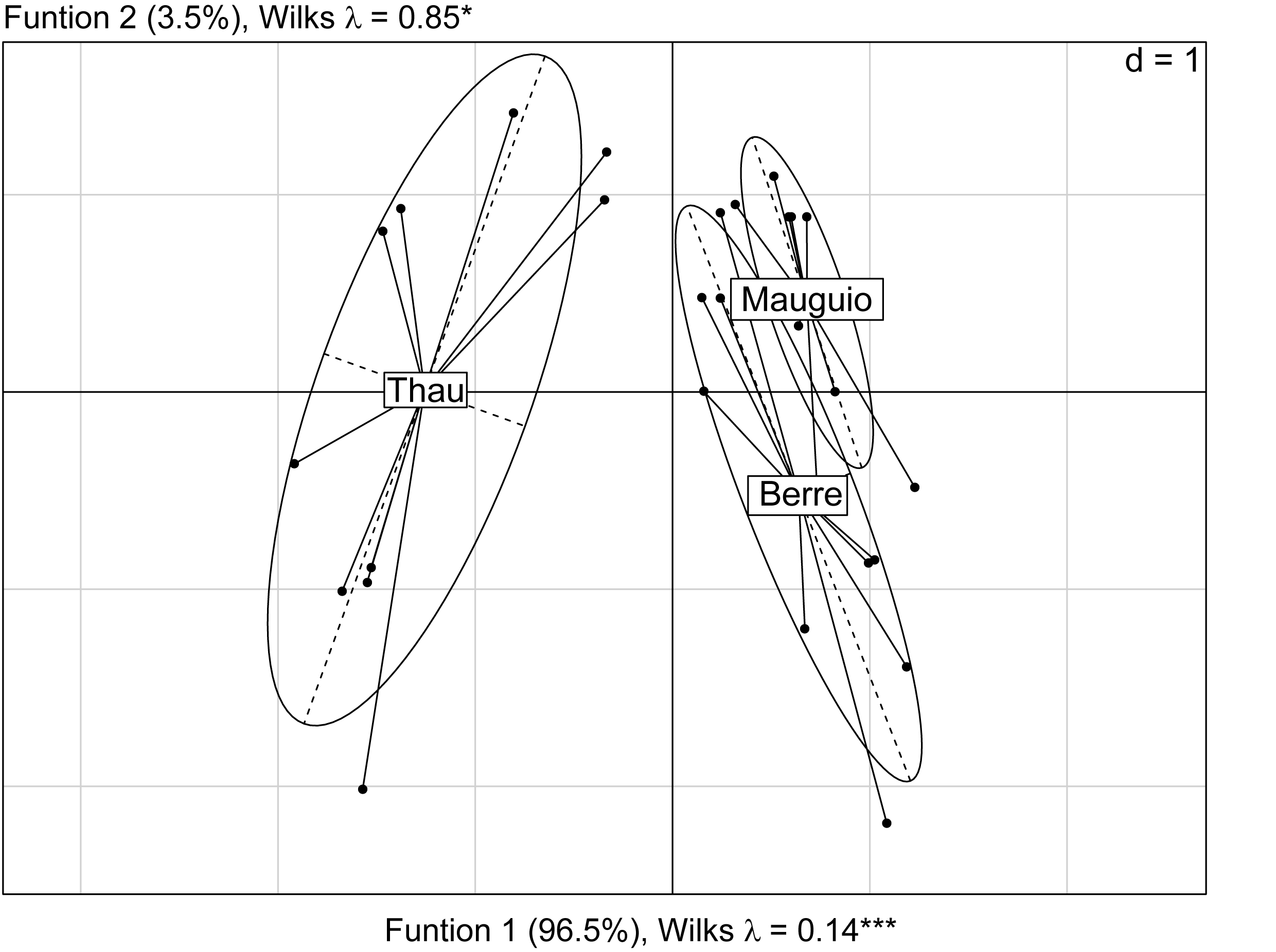

Supplement: Figure S1 — Canonical discriminant analysis performed with Sr:Ca and Ba:Ca of fish collected in nurseries in order to evaluate the difference between nurseries (in 2008). Function 1 and 2 are linear combinations of descriptors that maximize the Wilks λ. Each function represents a part of the total variability (in %) of dataset. The Wilks λ allows assessment of the performance of the discriminant analysis. The values of λ range from 0 to 1, and the closer the λ is to 0, the better is the discriminating power of the CDA. (TIF) [file pone.0086585.s001.tif]

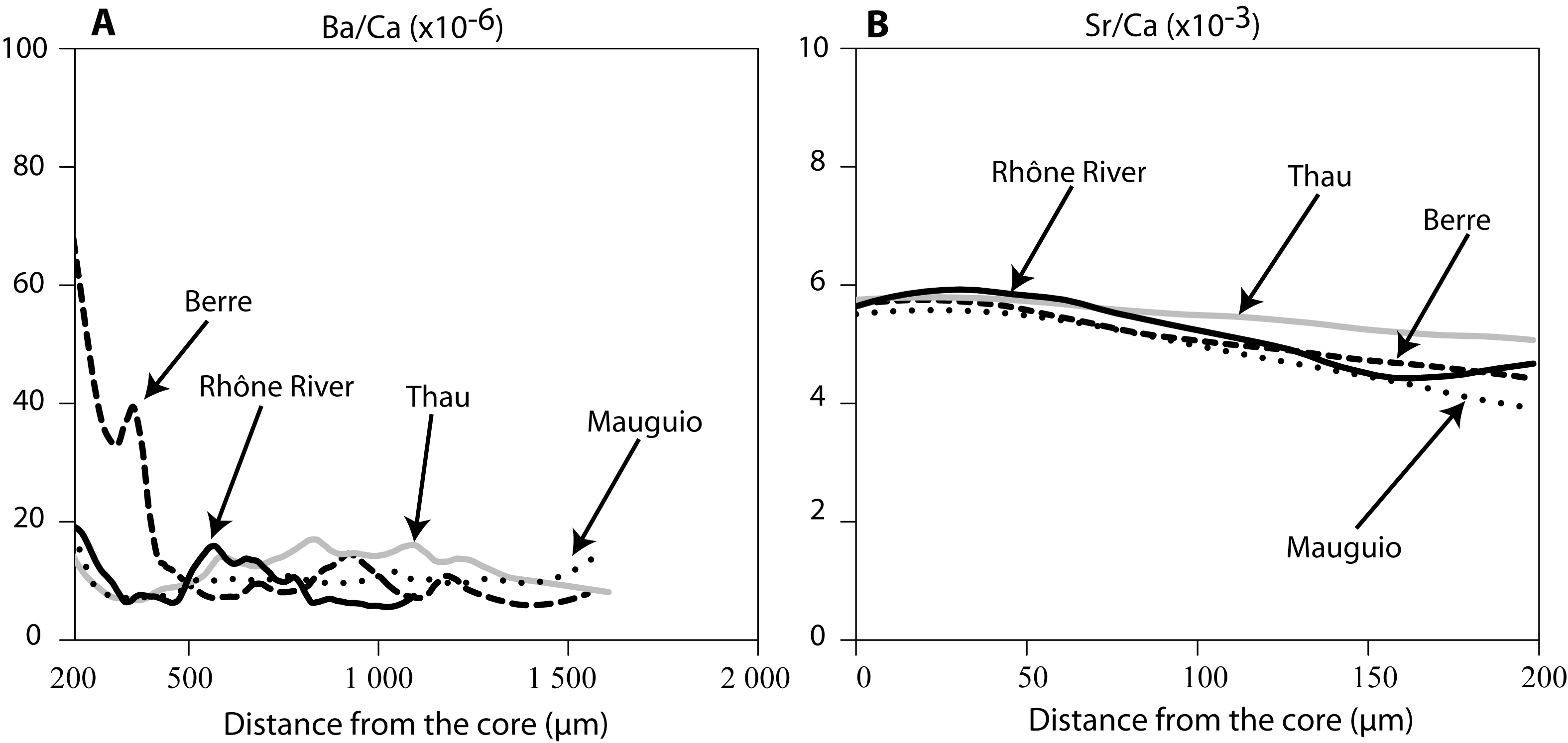

Supplement: Figure S2 — Mean Ba/Ca and Sr/Ca ratios of YOY from nurseries. Ba/Ca ratios are for the juvenile stage (A) whereas Sr/Ca ratios are for the larval stage (B). (TIF) [file pone.0086585.s002.tif]

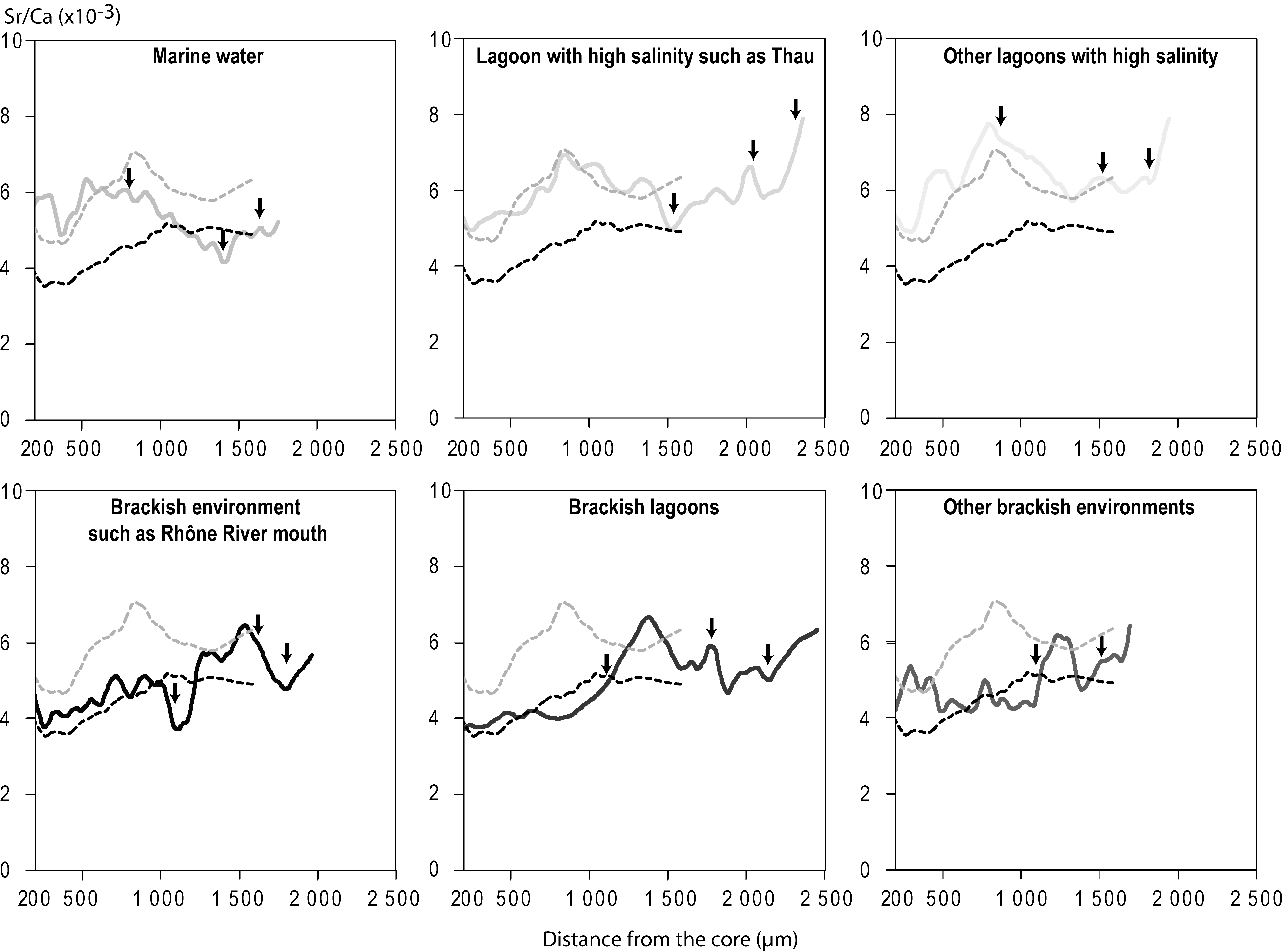

Supplement: Figure S3 — Example of comparison between individuals from the 6 potentials nursery types inhabited by soles during the juveniles stage (solid lines) and type curve of nurseries with high salinity (grey dashed line) and brackish environments (dark dashed line). The arrows represent the annual marks observed in adult profiles. Common soles leave nurseries at the end of the first year of life, thus the comparisons between potentials types of nurseries and adults profiles were performed only for this period. (TIF) [file pone.0086585.s003.tif]
