# Supplementary material for: The Great Melting Pot. Common Sole Population Connectivity Assessed by Otolith and Water Fingerprints
Source: PLoS One. 2014 Jan 27;9(1):e86585. doi: 10.1371/journal.pone.0086585 (PMC3903582; doi:10.1371/journal.pone.0086585)
Supplement: File S1 — Includes Tables S1 - S3. Table S1. Results of MANOVAs performed with Sr:Ca and Ba:Ca ratios of fish collected in nurseries in order to evaluate the variations over time and the differences between nurseries. Table S2. Mean (±sd) and results of t-test performed on the Sr:Ca and Ba:Ca measured on the larval and juvenile life stages of YOY from Mauguio in 2004 and 2008. Table S3. Seasonal mean (±sd) of the daily increment width of otoliths from YOY collected in nurseries and results of comparisons between nurseries (ANOVAs and Fischer LSD). (DOC) [file pone.0086585.s004.doc]

Files S1 legend

Files S1 contains Table S1 to Table S3. Table S1: Results of MANOVAs performed with Sr:Ca and Ba:Ca ratios of fish collected in nurseries in order to evaluate the variations over time and the differences between nurseries. Table S2: Mean (±sd) and results of t-test performed on the Sr:Ca and Ba:Ca measured on the larval and juvenile life stages of YOY from Mauguio in 2004 and 2008. Table S3: Seasonal mean (±sd) of the daily increment width of otolith from YOY collected in nurseries and results of comparisons between nurseries (ANOVAs and Fischer LSD).

Table S1: Results of MANOVAs performed with Sr:Ca and Ba:Ca ratios of fish collected in nurseries in order to evaluate the variations over time and the differences between nurseries.

|  | MANOVA F | Post-hoc elemental differences | |
| --- | --- | --- | --- |
| Inter-annual comparisons |  |  |  |
| Thau | 8.83* | 2003=2004<2008 | Sr:Ca, Ba:Ca |
| Mauguio | 4.45* | 2008<2004 | Sr:Ca |
| Inter-sites comparisons |  |  |  |
| 2004 | 6.77** | Mauguio<Thau | Sr:Ca |
| 2008 | 23.10*** | Berre=Mauguio<Thau | Sr:Ca |
|  |  | Mauguio=Thau<Berre | Ba:Ca |

Table S2: Mean (±sd) and results of t-test performed on the Sr:Ca and Ba:Ca measured on the larval and juvenile life stages of YOY from Mauguio in 2004 and 2008.

| **Life stage** | **2004** | **2008** | **N** | **t** |  |  |
| --- | --- | --- | --- | --- | --- | --- |
| Larval life |  |  |  |  |  |  |
| Sr:Ca | 5.36±1.34 | 5.37±0.67 | 20 | -0.02 | ns |  |
| Ba:Ca | 34.18±35.12 | 33.78±49.03 | 20 | 0.02 | ns |  |
| Juvenile life |  |  |  |  |  |  |
| Sr:Ca | 4.78±0.42 | 4.33±0.21 | 20 | 3.03 | p<0.05 | 2008<2004 |
| Ba:Ca | 9.88±2.23 | 10.40±4.07 | 20 | -0.36 | ns |  |

*Comments: the comparison of fish elemental signatures of YOY soles collected in Mauguio in 2004 and 2008 showed no differences for the larval life stage and higher Sr:Ca ratios in 2004 for the juvenile stage. At the same time, mean values of Sr:Ca ratios during the juvenile life stage were distinctly lower (between 4.33 to 4.78) than those observed in fish from Thau (between 5.14 to 7.23). These results suggest a relative homogeneity of characteristics of the lagoons in this study despite some degree of interannual variability.*

Table S3: Seasonal mean (±sd) of the daily increment width of otolith from YOY collected in nurseries and results of comparisons between nurseries (ANOVAs and Fischer LSD).

| Season | Thau (T) | Mauguio (M) | Berre (B) | Rhône (R) | ANOVA F | Post-hoc |
| --- | --- | --- | --- | --- | --- | --- |
| Winter | 3.53±1.49 | 4.76±2.09 | 4.17±2.44 | 4.21±2.16 | 103.2*** | T <R=B<M |
| Spring | 3.69±1.57 | 5.13±2.24 | 3.69±2.12 | 4.76±1.89 | 483.5*** | T=B<R<M |
| Summer | 3.93±1.43 | 5.55±2.34 | 4.90±2.24 | 5.53±1.89 | 492.0*** | T<B<R=M |
| Fall | 4.14±1.63 | 5.95±3.14 | 7.19±2.88 | - | 598.0*** | T<M<B |
